# Identification of cells of leukemic stem cell origin with non-canonical regenerative properties

**DOI:** 10.1016/j.xcrm.2024.101485

**Published:** 2024-04-05

**Authors:** Cameron G. Hollands, Allison L. Boyd, Xueli Zhao, Jennifer C. Reid, Charisa Henly, Amro ElRafie, David Boylan, Emily Broder, Olivia Kalau, Paige Johnson, Alyssa Mark, Jamie McNicol, Anargyros Xenocostas, Tobias Berg, Ronan Foley, Michael Trus, Brian Leber, Alejandro Garcia-Horton, Clinton Campbell, Mickie Bhatia

**Affiliations:** 1 Michael G. DeGroote School of Medicine, Department of Biochemistry and Biomedical Sciences, McMaster University, Hamilton, ON L8N 3Z5, Canada; 2 Department of Medicine, Division of Hematology, Schulich School of Medicine, University of Western Ontario, London, Ontario N6A 3K7, Canada; 3 Hematology Exploration and Applications in Leukemia (HEAL) Program, Hamilton, ON, Canada; 4 Department of Oncology, McMaster University, Hamilton, ON L8S 4L8, Canada; 5 Department of Pathology and Molecular Medicine, McMaster University, Hamilton, ON L8S 4L8, Canada

**Keywords:** regeneration enriched cells, non-canonical regeneration, acute myeloid leukemia, injury, chemotherapy, Regen71, relapse, leukemia stem cells

## Abstract

Despite most acute myeloid leukemia (AML) patients entering remission following chemotherapy, outcomes remain poor due to surviving leukemic cells that contribute to relapse. The nature of these enduring cells is poorly understood. Here, through temporal single-cell transcriptomic characterization of AML hierarchical regeneration in response to chemotherapy, we reveal a cell population: AML regeneration enriched cells (RECs). RECs are defined by CD74/CD68 expression, and although derived from leukemic stem cells (LSCs), are devoid of stem/progenitor capacity. Based on REC *in situ* proximity to CD34-expressing cells identified using spatial transcriptomics on AML patient bone marrow samples, RECs demonstrate the ability to augment or reduce leukemic regeneration *in vivo* based on transfusion or depletion, respectively. Furthermore, RECs are prognostic for patient survival as well as predictive of treatment failure in AML cohorts. Our study reveals RECs as a previously unknown functional catalyst of LSC-driven regeneration contributing to the non-canonical framework of AML regeneration.

## Highlights

• Single-cell transcriptomics of AML PDXs during chemo-induced regrowth reveals RECs
• Patient-derived RECs are devoid of stem cell capacity but predict patient outcome
• Spatial transcriptomics of AML BM biopsies reveal proximity of RECs to primitive cells
• Functional analyses reveal RECs non-canonically catalyze leukemic regeneration

## Graphical abstract

**Figure:**
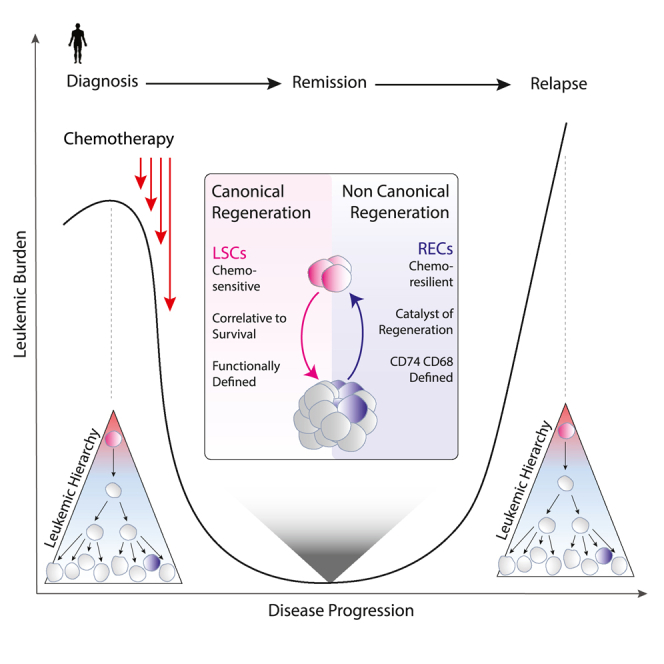

## Teaser

Hollands et al. discover a subset of acute myeloid leukemia cells termed regeneration enriched cells (RECs). RECs do not have stemness properties, but functionally catalyze leukemic regeneration *in vivo* post chemotherapy. This causal role provides evidence for non-canonical leukemic regrowth as a potential biomarker or therapeutic target in preventing relapse.

## Introduction

Similar to other stem cell-driven cancers, acute myeloid leukemia (AML) cells are assembled in a hierarchy. AML represents an aggressive and heterogeneous hematological cancer characterized by a block in differentiation that affects myeloid lineages of the hematopoietic tissue.^1^^,^^2^ This results in an accumulation of poorly differentiated blast cells in the patient bone marrow (BM).^2^ From a treatment perspective, the major barrier in achieving disease-free survival of AML patients is maintaining a state of clinical remission (CR) defined by less than 5% blasts^3^^,^^4^ and thus preventing disease regrowth above this threshold. Subsets of leukemic cells remain and survive in the BM post chemotherapy that are capable of reinitiating disease and giving rise to relapsed AML responsible for high mortality rates.^5^ The nature, properties, and overall dynamics of the enduring cells that contribute to and are responsible for the AML regeneration processes are poorly understood. Altogether, this has limited consistent biomarker detection to better manage patients post chemotherapy or to develop novel targeted therapies for AML relapse.

Residual leukemic cells responsible for regeneration of disease post chemotherapy are believed to be enriched for leukemic stem cells (LSCs) and define the basis of “canonical AML regeneration” contributing to relapsed disease. LSCs are considered to be at the cellular apex of this hierarchically arranged malignant tissue, and are operationally defined by the ability to engraft and initiate patient-specific leukemia upon transplant into immune-deficient mice.^6^^,^^7^ Features associated with LSCs are quiescence and self-renewal, which supports the notion that LSCs can evade the anti-proliferative chemotherapy by maintaining a dormant state,^8^ and subsequently drive canonical regeneration. As *in vivo* engraftment tests are laborious and retrospective, molecular surrogate definitions of LSCs using complex gene profiling have been devised.^9^^,^^10^ The LSC-R^9^ and the weighted gene profile of LSC17^10^ were defined by their ability to correlate to engraftment activity and overall survival (OS) of AML patients, respectively.^9^^,^^10^ However, findings of recent studies using *in vivo* xenograft models have suggested the involvement of elements other than LSCs in disease regrowth, thereby transcending the canonical concept of AML regeneration. These studies mimicked the clinical treatment of AML in xenograft models and all observed reductions in LSC numbers and quiescence following chemotherapy,^11^^,^^12^^,^^13^ providing collective evidence that properties of self-renewing cells, quiescent cells, and immunophenotypically primitive cells are in fact depleted following chemotherapy treatment in patients, patient-derived xenograft (PDX) models, and *in vitro* models of AML.^11^^,^^12^^,^^13^ These observations suggest non-canonical alternative mechanisms contribute to the complexity of AML regeneration^12^^,^^14^ and underscore the need for further characterization into the complex nature of AML regeneration post chemotherapy treatment vs. naive LSC states.

To date, the cellular and molecular basis of AML regeneration post chemotherapy has yet to be sufficiently resolved to combat disease relapse. We propose that the cellular processes of AML regeneration are not only complex, but temporally dynamic in nature, including changing molecular and cellular profiles in response to chemotherapy. In this case, biological processes would be best captured by evaluating multiple time points of regeneration using detailed molecular analyses paired with functional assays shortly after chemotherapy treatment. Temporal analysis using human-mouse PDX models and single-cell transcriptomics has revealed a subset of non-stem/progenitor accessory cells that act as a catalyst for AML regeneration with functional and clinical prognostic properties that contribute to non-canonical framework of AML regeneration operating in conjunction with LSCs.

## Results

### Temporal analysis of AML PDXs following chemotherapy captures cellular dynamics of disease regeneration

**Figure 1:**
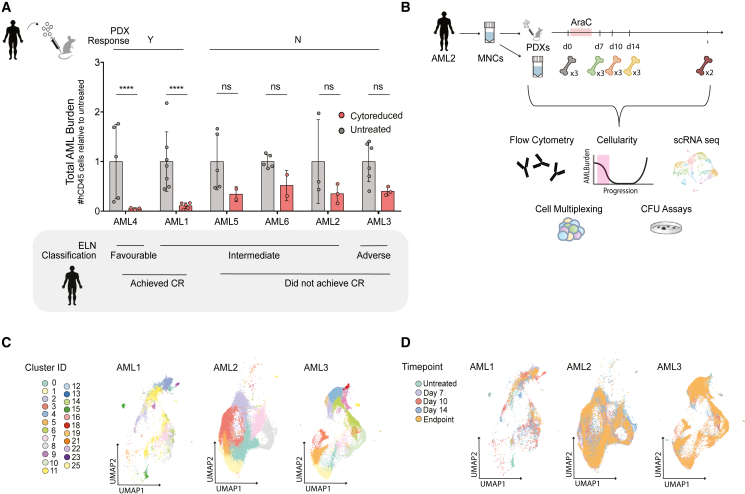
scRNA-seq data on frequently sampled AML xenografts following cytarabine treatment identifies unique kinetics and defines both responding and non-responding PDXs (A) Disease burden (hCD45Chimerism ∗ Total Cells Harvested) represented by mean ± SD from PDXs at day 0 (“Untreated”) and at the lowest disease burden time point (“Cytoreduced”) normalized to Untreated AML1-6, sorted by ELN stratifications, clinical outcome, and responder status (N = 6, n = 31 untreated, n = 18 treated). (B) Experimental overview of data generating panels (C) and (D): *De novo* patient tissue and hCD45^+^ BM harvested from three patient-matched PDXs at each noted time point underwent scRNA-seq with cell multiplexing, immunophenotyping, functional assays, and cellularity assessments. UMAP plots of cells from all time points of AML1-3 PDXs organized by (C) transcriptionally defined cell cluster ID and by (D) time point. ∗∗∗∗p < 0.0001, ∗p < 0.05, ns p > 0.05 by unpaired t tests. See also Figure S1 and Table S1.

Cytarabine (AraC) is the backbone of the standard of care in AML treatment^4^^,^^15^ and has been used to model chemotherapy treatment in xenografts,^11^^,^^12^^,^^13^ as other agents given to AML patients, such as daunorubicin, are toxic in xenografts.^13^ AraC is a pyrimidine nucleoside analog and functions in a non-specific fashion to target highly proliferative cells.^16^ We modeled AraC treatment through a 5-day administration mimicking clinical use in patients, as shown previously^13^ in AML PDX initiated from six patients with diverse European Leukemia Network (ELN)^1^ stratification (AML1-6, Table S1). Once AML disease was established in recipient mice, the dynamics of AML response were analyzed at three sequential time points post treatment (days 7, 10, and 14). Disease burden (total number of AML cells [hCD45^+^% ∗ total harvested cells]) was analyzed throughout the follow-up time points post chemotherapy treatment in PDXs derived from all patient samples (Figures 1A and S1A). The dynamics of these changes were variable across patients, consistent with our expectations that multiple time points of analysis are required to capture the true cellular kinetics of AraC-induced cytoreduction and subsequent regeneration. Absolute PDX response to chemotherapy was patient specific (p < 0.05, two-way ANOVA) and supported the separation of patients into categories of responders vs. non-responders based on the disease burden in the AML PDXs: responders being statistically reduced (PDXs: AML1, AML4) and non-responders being non-statistically reduced (PDXs: AML2-3, 5–6) following AraC treatment (Figure 1A). This distinction correlated to AML patient outcomes, where PDX responders entered CR after induction treatment, while PDX non-responders failed to enter induced CR, which broadly correlated with predicted outcome based on ELN classification (Figure 1A). An alignment of clinical metrics to chemosensitivity in PDX models has not been reported in experimental settings to date, and possibly observed here due to maximized chemosensitivity time points compared among patients. This is exemplified in AML patient 1 PDXs, where a single follow-up at day 10 would not capture the acute cytoreduction shown at day 7 and would otherwise be incorrectly classified as a non-responder (Figure S1B).

With kinetics of AML regeneration biologically established *in vivo*, we used this foundational data to understand the molecular dynamics of AML cells during regeneration. Using droplet-based single-cell RNA sequencing (scRNA-seq), we generated libraries from non-responder (AML2 and AML3, ELN intermediate and adverse, respectively) and responder (AML1, ELN intermediate) PDXs at days 0, 7, 10, and 14 post chemotherapy treatment, as well as at the ethical endpoint of each recipient xenograft (summarized and depicted in Figures 1B, S1D, and S1E). To ensure AML-specific features could be distinguished, we performed parallel experiments using healthy hematopoietic stem cells (HSCs) from umbilical cord blood (CB) engrafted in PDX mice to obtain healthy control cells before and after chemotherapy treatment *in vivo* (Figure S1F). We merged a total of 42 resulting PDX samples used for scRNA-seq by batch correcting and integrating using the Seurat integration package,^17^ with healthy BM scRNA-seq data as a reference anchor (Figure S1C). Resulting individual cell gene expression could be summarized by stratification into 26 transcriptionally defined cell clusters (Figure 1C). Greater variability of clusters throughout time points post chemotherapy was qualitatively observed in AML1 PDXs (responders) as compared with AML 2 and 3 PDXs (non-responders) (Figure 1D). To provide quantitative temporal evaluation for each cluster over time, a metric of cluster volatility was utilized: 1 – (Lowest % over time/highest % over time) for each shared cluster from all three AML patients (Figure S1G). Cluster volatility over time was found to be significantly higher in the responding AMLs as compared with the non-responding AMLs, indicative of greater transcriptional change in AML disease cells that effectively respond to therapy, whereas non-responsive disease reflects reduced transcriptional changes accompanied with disease retention. These analyses provide a basis to dissect graded responses of AML disease to chemotherapy and transcriptionally define potential cell entities involved in the dynamic destruction and subsequent regeneration of the human AML hierarchy.

### Identification of transcriptionally assigned cells that correlate to functional states of regeneration in PDX models: Regeneration enriched cells

AML disease is suggested to be organized in a hierarchy sustained by LSCs at its apex. This same disease hierarchy can be established in immunodeficient mice by leukemic initiating stem cells upon transplantation of cells from AML patients, and the resulting surrogate murine recipient can be treated with chemotherapy.^11^^,^^12^^,^^13^ Aside from level of leukemic burden measured in PDX models by amount of human leukemic cells, the activation state of leukemic regeneration was shown to faithfully be represented by leukemic progenitor activity.^13^^,^^18^ Accordingly, both leukemic burden and progenitor activity were used as metrics to identify the biologically relevant time points of (1) cytoreduction and (2) subsequent regenerating AML (Figure S2A).

**Figure 2:**
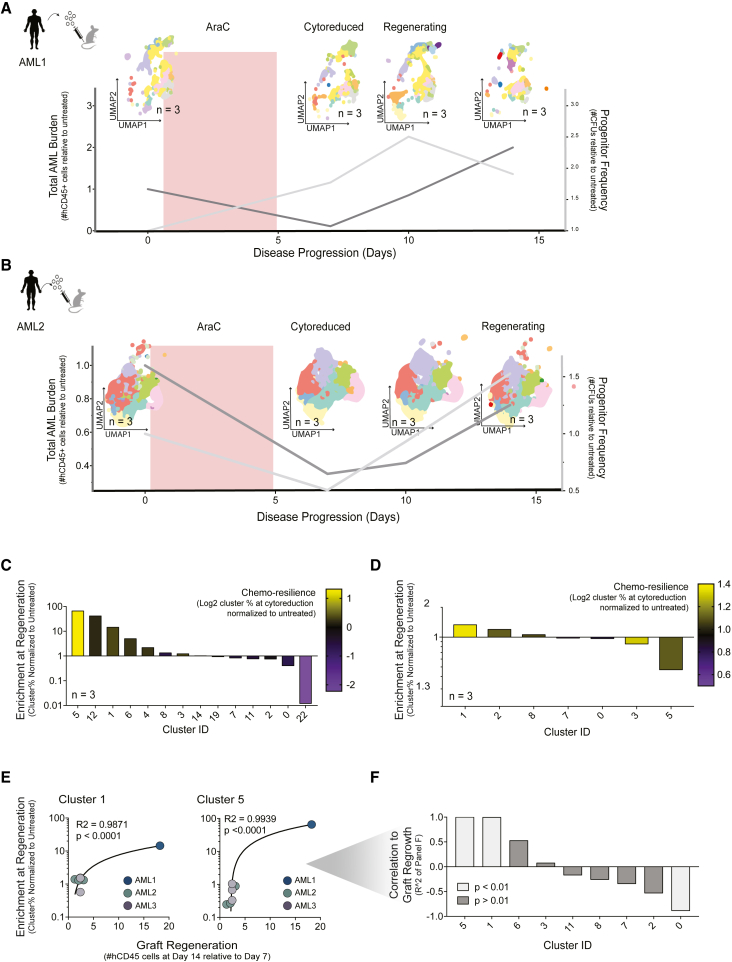
Cluster 5 and cluster 1 are most enriched at functionally defined biologically relevant time points in AML1 and AML2, respectively (A and B) Disease burden (Total cells harvested ∗ %hCD45; dark gray) and progenitor frequency (#colonies/cells seeded; light gray) during and following a 5-day AraC treatment in (A) AML1 PDXs and (B) AML2 PDXs, overlayed with UMAP plots of scRNA-seq data (n = 3 per time point per AML sample [AML1 pooled]) from the same cell pool that derived the functional progenitor frequency and disease burden data throughout the time course. (C and D) Bar graphs of the fold enrichment of each substantive cluster at untreated vs*.* regeneration time points for AML1 (n = 3, pooled) and AML2 (n = 3). Color of bars represents a metric of cytoreduction magnitude [Log10(Cluster% Untreated/Cluster% Cytoreduced)]. (E) Correlation plots between fold enrichment of clusters 1 and 5 at untreated vs*.* regeneration time point (Cluster% at Regeneration/Cluster% at Untreated) compared with fold increase of total AML cell burden at day 7 to day 14. (F) Bar graph of the correlation coefficient (*R*^2^ value) from linear regression analysis from (E) from all shared and substantive clusters. The linear regression reveals a correlation that is significantly non-zero (p > 0.01, light gray bars) or not significant (p > 0.05, dark gray bars). See also Figure S2, Tables S1 and S2.

*In vivo* biological readout of AML disease was matched to single-cell transcript profiling derived from the same PDX for each time point to identify transcriptomic patterns and clusters associated with AraC resilience and leukemic hierarchical regeneration. Using deep functional and scRNA-seq analysis, AML1 and AML2 were selected as representative responding and non-responding patients, respectively. The greatest disease burden reduction was identified at day 7, signifying the lowest threshold of cytoreduction, whereas peak leukemic AML progenitor frequency signifying regeneration, was detected at day 10 in the responder and day 14 in the non-responder (Figures 2A and 2B, S2B–S2E). Nearly all clusters were depleted following chemotherapy exposure after incorporating a disease burden metric (cluster proportion ∗ total AML cells harvested; Figure S2F) based on three PDX recipient mice for each time point for each patient (Figures 2A, 2B, and S2F). Similar results were obtained using unbiased categorization methods to assign cell type assignment to each cell,^19^ which also revealed each cell type was diminished following chemotherapy (Figure S2G). Collectively, these results indicate that cytoreductive chemotherapy targets the complete array of cells of the AML hierarchy without prejudice. Despite cytoreduction of AML cells in response to chemotherapy, clusters with the least reduction after chemotherapy were identified in cluster 5 in AML1 and cluster 1 in AML2 and these same clusters were also the most enriched at corresponding regeneration time points (Figures 2C and 2D). Cluster 5 was preferentially enriched at functional regeneration by 68-fold in responding AML (Figure 2C, n = 3), whereas cluster 1 from the non-responding AML was enriched by only 1.3-fold (Figure 2D, n = 3). To avoid artifacts from single PDXs, each time point consisted of three pooled PDX biological replicates and confirmed that cluster dynamics were consistent across pooled biological replicates by cell multiplexing analysis (Figure S2H). Cluster 1 was highly prevalent before and after chemotherapy in AML2 PDX recipients, consistent with the expected dampened response to chemotherapy in non-responders. In contrast, cluster 5 emerged almost exclusively during AraC treatment in chemotherapy responsive AML patient xenografts.

Given the correlation of these clusters with AML regeneration kinetics *in vivo*, we termed clusters 1 and 5 as regeneration enriched cells (RECs). Transcriptionally, RECs resemble non-cycling monocyte-like profiles (Figures S2I and S2J). Gene expression profiles of RECs (Data S1) showed enrichment of proinflammatory and oxidative phosphorylation profiles by gene set enrichment analysis (GSEA) of HALLMARK, REACTOME, WIKI, and KEGG pathways (Figure S2K), a feature previously observed in cytarabine response and AML disease regrowth.^11^^,^^12^^,^^20^^,^^21^ To explore the applicability of REC clusters 1 and 5 to functional AML regeneration, we expanded our analysis to an interpatient level to include all PDXs with paired scRNA and functional datasets, AML1-3 (Figures 1A and S1E; N = 3, n = 7). Fold enrichment of REC clusters directly correlates to the magnitude of graft regrowth, as measured by total leukemic cells from day 7 to day 14 (Figure 2E). Even though REC clusters were derived from distinct chemotherapy responses across patients, our findings show consistent correlation between emergence of RECs in all AML PDXs with functional leukemic growth. Using similar analysis, no other clusters were found to positively correlate to the magnitude of AML graft regeneration (Figure 2F). The magnitude of REC cluster emergence as a direct function of AML graft regrowth aggressivity suggests RECs may represent unique cell types involved in AML regeneration.

### RECs in patient-derived diagnostic tissue predict clinical outcomes and are immunophenotypically defined by CD68/CD74 expression

**Figure 3:**
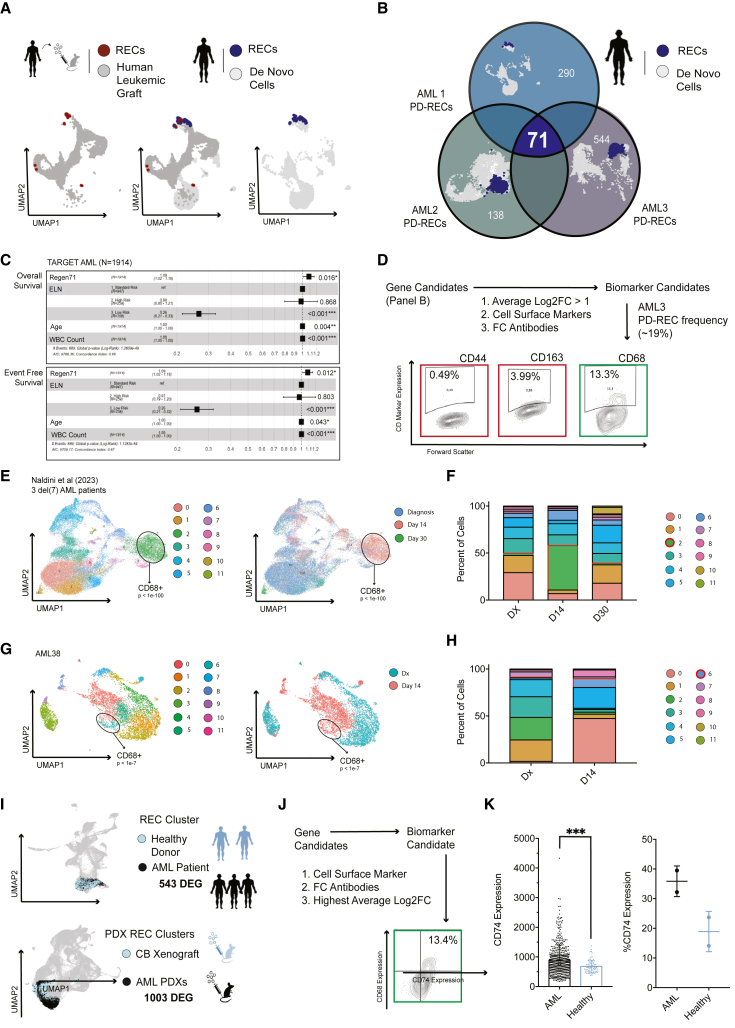
REC gene expression demonstrates predictive capacity of AML patient survival, and RECs are defined by the CD74^+^/CD68^+^ immunophenotype (A) Patient-specific UMAP plots of all PDX and *de novo* cells from AML1 highlighting RECs from PDXs (dark red) from *de novo* tissue (dark blue). (B) The 71 shared DEGs that overlap between RECs of AML1-3. (C) Multivariate cox regression analysis on Regen71, ELN stratifications, age, and WBC count with overall and event-free survival of an independent AML cohort (TARGET-AML, N = 1,914). (D) Flow of narrowing down the Regen71 gene score to CD68 as a biomarker for RECs. (E) UMAP plots of cells from del(7) AML patients throughout treatment time course (N = 3)^25^ with CD68-enriched cluster 2 highlighted, grouped by cluster ID and clinical time point, respectively. (F) Bar plot of cluster composition of each time point of scRNA from (E). CD68^+^ cluster 2 highlighted in red emerged post chemotherapy. (G) UMAP plots of *de novo* AML38 cells at diagnosis and when refractory to treatment with CD68-enriched cluster 6 highlighted, grouped by cluster ID and treatment time point, respectively. (H) Bar plot of cluster composition of each time point of scRNA from (G). CD68^+^ cluster 6 highlighted in red emerged post chemotherapy. (I) UMAP plots of *de novo* REC cluster 0′ colored by tissue source (healthy BM: n = 1,123, AML sample: n = 2,696) and of PDX REC clusters 1 and 5 colored by tissue source (CB xenograft: n = 2,305, AML PDX: n = 9,403). (J) Logic flow of narrowing down the shared DEG from (G) to CD74 as a biomarker for leukemia-specific RECs. (K) LogCD74 expression by RNA-seq represented by mean ± SD of AML patients (N = 542) compared with healthy BM cells (N = 73) from leukemia MILE study (∗∗∗p < 0.001, Student’s t test), and CD74 expression by FC on AML patients (N = 2) compared with healthy BM donations (N = 2). See also Figure S3, Tables S1 and S2.

To evaluate the relevance of PDX-generated REC profiles (cluster 1 or 5), we examined whether RECs could be observed in AML patients. scRNA-seq analysis was applied to *de novo* samples of AML patients 1, 2, and 3, and scRNA-seq data were integrated and batch corrected with patient-matched PDX scRNA-seq data for all AML xenograft time points used (n = 39).^17^ This was performed on a per-patient basis to reduce variability that may arise from inter-patient heterogeneity.^1^^,^^22^ In all AML patients examined, RECs previously identified in PDXs were highlighted (Figures 3A and S3A dark red) and primarily assigned to a single cluster for each AML patient. Transcriptionally similar cells from *de novo* sources were present within these clusters (Figures 3A and S3A dark blue), indicating on a transcriptional level the RECs are present in patients at diagnosis. The ability to identify PDX-derived RECs as a transcriptionally definable entity in AML patients demonstrates that RECs are not a generated artifact of AraC treatment or PDX modeling.

To characterize REC properties potentially shared among AML patients, we identified differentially expressed genes (DEGs) of *de novo* sourced RECs (Figure 3B, Data S1) compared with all *de novo* cells from each AML patient. This revealed a total of 71 DEGs that were common among AML1-3, which we termed the Regeneration-71 score (Regen71) (Figure 3B, Data S1). To be thorough and to ensure we are capturing the same biological phenomena in all three patients, *de novo* scRNA datasets from AML patients 1 to 3 were merged with two non-leukemic BM donor samples (BM1 and BM2) and re-clustered to best capture interpatient variability (Figure S3B). Accordingly, cluster numbers from this dataset were denoted with a prime symbol to differentiate from PDX datasets (Figures 1, 2, and 3A); cells from all samples could be stratified into clusters 0′–17′ (Figure S3B). Previously identified RECs from AML patients 1, 2, and 3 at regeneration cluster together within newly assigned cluster 0′ (Figure S3B). Cluster 0′ hosted 653 upregulated genes (Data S1), which contained the previously identified Regen71.

The Regen71 is a gene score that represents the biological process of leukemic regeneration. Based on this and its absence of primitive gene expression (Figures S2I–S2K), this likely represents a distinct biological entity other than AML LSCs. To examine the value of the Regen71, we performed multivariate cox regression analyses on three publicly available gene expression datasets,^23^ separately accounting for both comparable molecular scores (LSC-R,^9^ LSC17,^10^ Data S1) and clinical covariates (age, ELN stratifications, white blood cell [WBC] count). In the TARGET-AML cohort (N = 1914), the Regen71 correlated to both OS and event-free survival (EFS) (Figures 3C and S3C). When filtering the cohort for patients who received the same induction treatments, prognostic capabilities improved (Figure S3C). In the TCGA-LAML cohort (N = 151), the Regen71 also correlated with OS in the molecular analysis and approached significance in the clinical analysis (Figure S3D). The Metzeler et al.^23^ (N = 79) patient cohort had none of the gene scores achieve significance, although the LSC17 and Regen71 approach it (Figure S3D). In addition, the TARGET-AML cohort was subdivided into the three ELN stratifications and the molecular multivariate analysis performed revealed Regen71 to be the most predictive in the ELN high-risk group (Figure S3E). Notably, the Regen71 shows predictability of EFS and OS in both pediatric and adult AML, indicating this gene score may be independent of aging processes. Thus, the Regen71 provides a straightforward, and unweighted signature of genes to evaluate the dynamics of human AML regeneration that have not been explored to date and justifies further investigation into this score and related biological processes.

We next aimed to use Regen71 gene profile to identify a putative REC immunotype, unlike LSC17 or LSC-R. Genes encoding for putative cell surface markers of RECs were identified and using the criteria of gene expression strength (Average Log2 FC > 1) and commercially available well-established flow cytometry (FC) antibodies. CD68, CD163, and CD44 were revealed as candidate biomarkers (Figure 3D). To ensure gene expression of candidates translated to protein expression, we cross-referenced positive population frequency by FC on AML3 cells and compared the frequency of *de novo* AML3 REC cluster (Figures S3A and S3F) within scRNA-seq data on AML3 (19%). CD68 was closest to the frequency at 13.3%, while the CD163 and CD44 (Figure 3D) failed to achieve precise cellular identity. Similarly, in patient AML1, the proportion of the CD68 by FC reflected the proportion of the REC cluster in the scRNA-seq dataset (Figures 3A and S3F). Accordingly, we selected CD68 as a candidate biomarker for RECs.

To test whether the transient emergence of RECs is robust and reproducible in external cohorts, we surveyed an additional 14 AML patient datasets. In three del(7) AML patients from Naldini et al.,^24^ the CD68 enriched cluster (cluster 2) is temporally enriched 14 days following treatment initiation (Figures 3E and 3F). In another AML patient (AML38), a CD68 enriched cluster (cluster 6) expands after an unsuccessful treatment (refractory to treatment, Figures 3G and 3H). In a 10-patient NPM1 mutated AML cohort with paired NPM1 aberration mutational status,^24^ a CD68-positive cluster (cluster 1) is temporally enriched 14 days following chemotherapy initiation (Figures S3F and S3G). This dataset exhibited multiple CD68^+^ clusters wherein NPM1 mutational frequency varied (Figure S3G). Overall, the consistency of REC emergence in diverse patients from other studies post chemotherapy provides a promising pattern that necessitated further investigation.

As CD68^+^ populations with lower frequency of leukemic mutations were present in the NPM1^+^ AML 10-patient cohort^24^ (Figure S3H), we suggest that another biomarker should be included to deplete CD68^+^ cells of the healthy hematopoietic system from RECs. Using a *de novo* AML scRNA-seq dataset (Figure S3B), we performed DEG analysis comparing cells within REC cluster 0′ of AML1-3 with cells in REC cluster 0′ from healthy BM donors (Figure 3I). This revealed 543 enriched genes in the leukemic fraction of cluster 0′ (Data S1). Similar DEG analysis was performed on the PDX scRNA datasets (Figures 1C, 1D, and S1C), comparing the AML PDX-sourced cells and CB donor xenografts (clusters 1 and 5). A total of 1,003 genes were upregulated in the AML PDX vs*.* CB xenograft in clusters 1 and 5 (Data S1), consisting of a 420-gene overlap with AML-specific cells of cluster 0′ (Data S1). This revealed CD74, CD81, CD63, and CD47 as candidate cell markers, with CD74 having the highest Log2FC average value. The double-positive CD74/CD68 protein expression profile was still consistent with the expected concentration of patient-derived RECs of AML3 and is detectable as populations in newly diagnosed AML patients (Figures 3J and S4C, N = 25). We validated CD74 as leukemia-specific marker using RNA gene expression AML vs. healthy gene expression data accrued by the MILE study^21^^,^^25^ (N = 543, N = 73 AML Healthy BM respectively, p < 0.001 Student’s t test) and by FC (N = 2, for AML and healthy BM, Figure 3K). We moved forward with defining RECs by the CD74/CD68 immunophenotype. The combined REC vs. malignant cells with AML vs. healthy cells comparison analyses provided a robust method of cell enrichment that accounts for intra-patient heterogeneity and specificity to AML disease vs. healthy hematopoiesis to provide a candidate leukemic-enriching immunophenotype for RECs. These studies allowed a departure from cell cluster profiling used to define LSC17 and LSC-R, to cell entity based on cell surface markers leading to cell isolation to purify cells for causal and functional characterization of RECs.

### Prognostic value of RECs in AML patient survival and therapeutic response

**Figure 4:**
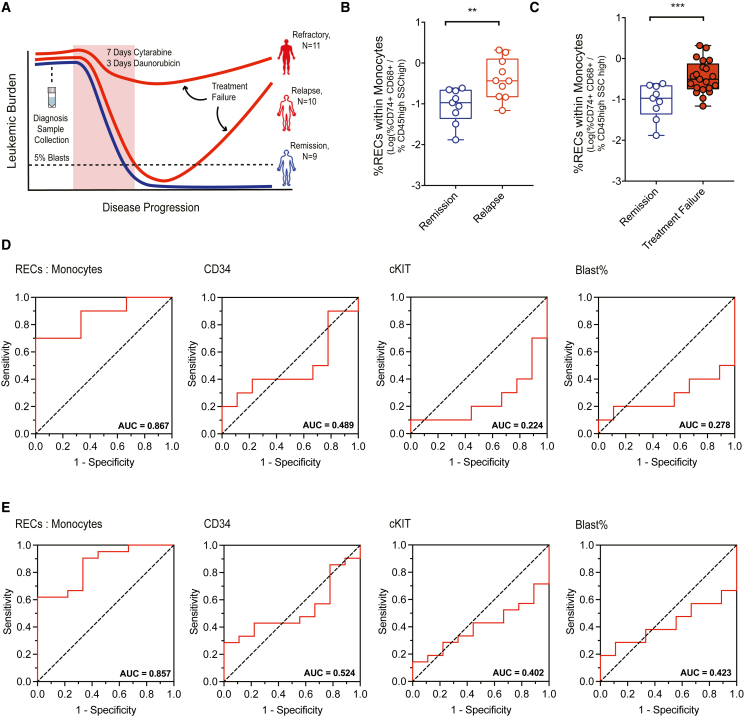
RECs demonstrate clinical potential (A) Visual representation of experimental design (N = 30). (B) Boxplots of %CD74/CD68 population relative to monocytic population (CD45^high^SSC^high^) of patients who entered remission (N = 9) compared with patients who relapsed after remission (N = 10). (C) The %CD74/CD68 population relative to monocytic population (CD45^high^SSC^high^) of patients who entered remission (N = 9) compared with patients who experienced either form of treatment failure (relapse or refractory, N = 21). ROC curves comparing the predictive capacity of (D) relapse and (E) treatment failure of %CD74/CD68:Monocytes ratio, CD34, cKit, and blast%. The greatest AUC value for both clinical outcomes was %CD74/CD68:Monocytes. (B) and (C) ∗∗∗p < 0.001, ∗∗p < 0.01 by unpaired Student’s t tests. See also Figure S4 and Table S1.

Given the prognostic value of RECs by gene expression (Regen71), we examined RECs defined by CD74^+^CD68^+^ cells in clinical management and treatment response of AML patients. Specifically, due to the derivation through association with functional regeneration in response to cytarabine treatment alone, we postulate that RECs may have clinical potential as a candidate relapse biomarker applicable to patients who were treated with standard 7 + 3 chemotherapy (cytarabine and daunorubicin). A biomarker detectable at diagnosis that predicts whether an AML patient will relapse after successful induction therapy would be a valuable clinical tool, as it could inform clinical decision making such as prompting follow-up rounds of consolidation therapy and hematopoietic stem cell transplants. REC frequency was analyzed using tissue samples from 30 independent AML patients from three distinct groups: Refractory to treatment (N = 11), entered CR but relapsed (N = 10), and entered long-term CR (N = 9) (Figure 4A). REC frequency (gating strategies: Figures S4C and S4D) successfully stratified those patients who remained in CR vs. those who eventually relapsed (p < 0.05, Figure S4A), demonstrating potential as a relapse marker detectable at diagnosis. RECs failed to stratify patients who suffered general treatment failure (both refractory and relapse) from patients who remained in remission (Figure S4B). As myeloid leukemias can occur at many stages of primitiveness within the leukemic hierarchy, including leukemias that are molecularly similar to RECs (monocytes), we hypothesized that normalizing REC percentage to the monocytic compartment percentage of each respective AML will relieve some complexity of interpatient heterogeneity. Based on standard monocytic CD45^bright^SSC^bright^ gates within all live cells (Figures S4D and S4E), the REC:monocyte ratio improved the prognostic value of RECs (Figure 4B) and expanded the prognostic capacity of RECs to stratify overall treatment failure from sustained remission (Figure 4C). Flow cytometric presentation of RECs was found to be heterogeneous between patients in either the blast gate vs. monocyte gate (SSC hCD45 gating, Figure S4F), and we suggest that RECs play a vital role in AML biology support independent of AML differentiation status. To examine the value of the REC:monocyte measurement, we conducted receiver operating characteristic (ROC) analysis. ROC represents a well-established methodology for assessing viability of a clinical diagnostic tool, as it takes into consideration false positive/negative rates and eliminates biases of arbitrarily drawn thresholds to produce an area under the curve (AUC) value that summarizes the utility of the assay. Using AUC values from ROC curves we found REC:monocyte ratio predicted both relapse (Figure 4D) and treatment failure (Figure 4E) better than blast%, and primitive marker expression (CD34 and cKit) in this 30-patient cohort. The REC:monocyte ratio had an AUC of 0.867 and 0.857 for predicting relapse and treatment failure, respectively, widely regarded as a strong predictive assay.^26^ Accordingly, our results indicate the REC immunophenotype has prognostic value for both OS and therapeutic response of AML patients.

### RECs are devoid of intrinsic stemness capacity but have *in situ* proximity to putative CD34^+^ LSCs

**Figure 5:**
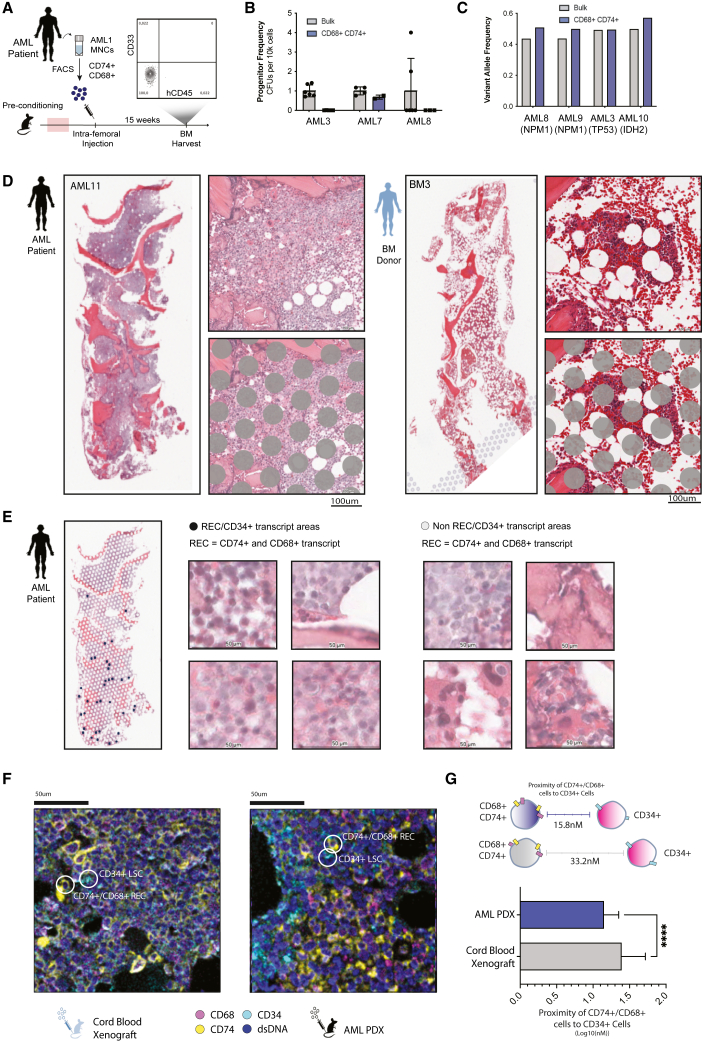
RECs demonstrate no stemness capacity and co-localize to CD34^+^ cells within leukemic tissue (A) Representative flow plot of hCD45 and CD33 expression in BM aspirates from PDXs 8 weeks post intra-femoral injection with RECs (n = 11, N = 3). (B) Bar graph of mean ± SD of CFU frequency (#colonies/cells seeded) of FACS-purified CD74^+^/CD68^+^ cells and bulk AML patient MNCs normalized to average AML patient MNCs. (C) Bar graph of leukemic mutation VAF of FACS-purified CD74^+^/CD68^+^ cells compared with control MNCs. (D) Whole H&E-stained tissue and representative images with and without spot overlays of BM tissue from AML patient 11 and BM donor 4. Scale bar, 100 μM. (E) Spots of CD74^+^/CD68^+^/CD34^+^ co-expression overlayed onto whole tissue section of AML BM11, and four representative images each from areas with and without CD74^+^/CD68^+^/CD34^+^ co-expression. Scale bars, 50 μM. (F) Xenograft BM sections of engrafted CB and AML, with hCD74 hCD68 hCD34 immunofluorescent labels by MIBI-TOF methodology. Examples of CD74^+^/CD68^+^ cells and CD34^+^ are highlighted in white. (G) Bar graph of the mean +/- SD distance between CD74+/CD68+ and CD34^+^ cells in the AML vs. CB xenograft BM (∗∗∗∗p < 0.0001, Student’s t test). See also Figure S5, Table S1.

To examine the relationship of RECs to LSCs, we performed FC analysis of RECs alongside conventional primitive leukemic cell immunophenotypes (CD34, CD90, CD117, CD123, CD38, and Tim3). Apart from Tim3, there is no enrichment of these markers on REC cell populations (Figures S5A and S5B), suggesting these RECs are a distinct biological entity. However, as features of stemness are defined functionally and not by correlative immunophenotypes, human CD74^+^CD68^+^ RECs were purified by fluorescence-activated cell sorting (FACS) and showed depleted LSC activity by either colony-forming unit (CFU) (progenitor) or xenotransplantation (LSC) assays (Figures 5A and 5B). To characterize the origin of RECs, patient-specific mutations were analyzed in purified RECs. These analyses demonstrated RECs are enriched for patient-specific leukemic mutations (N = 4, Figure 5C, Table S2) revealing human AML-derived RECs are of leukemic origin and are differentiation products of LSCs. Although dissimilar to LSCs by transcript, immunophenotype, and function, we observed an enrichment of REC high patients in LSC17 high patients compared to LSC17 low (Figure S5C), suggesting patients with greater RECs also contain greater numbers of LSCs. Consistently, within the TARGET-AML cohort, patients who have over the median score of Regen71 (REC^+^) and LSC17 (LSC^+^) have dismal EFS and worse ELN stratifications compared with REC^+^/LSC^−^, LSC^+^/REC^−^, and REC^−^/LSC^−^ patients (Figures S5D and S5E).

Based on these combined characteristic features of RECs, we postulated that RECs may serve as localized accessory cells to support leukemic stem/progenitor cell-driven disease regeneration. To examine this *in situ*, we used spatial transcriptomics analysis from four donated human trephine BM samples (two from AML patients vs. two from healthy donors). Tissues were H&E stained, and verified for expected differences between healthy donors and leukemic patients such as BM fat content^27^ and BM blast invasion (Figures 5D and S5F). This approach has each transcriptionally defined area of 50 μm in diameter (Figure 5D) and was used to investigate whether there are areas that contain both RECs and LSCs. We used CD68^+^/CD74^+^ transcript expression as a surrogate probe to define RECs in this analysis. LSC17 or LSC-R gene scores do not represent single-cell entities, but instead are powerful prognostic profiles and molecular definitions of putative LSCs derived through bulk cell assays and curated based on correlation to patient survival. As such, CD34 was chosen as a surrogate marker to represent LSCs and was shown to correlate to LSC17 signatures in previous studies.^18^

All tissues contained areas with CD74, CD68, and CD34 expression (Figure S5G) but only BM from AML patients had areas that overlapped CD74/CD68 and CD34 expression (Figure 5E). Representative images of REC/CD34^+^ proximity reveal consistency in tissue and morphology as compared with other regions of AML BM (Figure 5E). In sharp contrast, healthy BM sections were devoid of co-proximity of CD74^+^CD68^+^ and CD34^+^ cells. This suggested that CD74^+^/CD68^+^ cells may physically localize to CD34^+^ primitive cells in AML disease. To confirm that the proximity of RECs in AML patients was not a product of increased expression of markers within leukemic tissue, we examined the amount of CD74/CD68 expression within CD34^+^ and CD34^−^ areas. CD34^+^ areas had higher CD74 and CD68 expression as compared with CD34^−^ areas, while this pattern was absent in healthy tissues (Figure S5H), confirming a leukemia-specific proximity of RECs and CD34^+^ cells. Last, we wanted to ensure proximity was not due to CD34^+^ cell proliferation and differentiation into RECs and assessed the level of proliferation in relation to REC/CD34^+^ proximity. We have combined three transcript markers of cell cycle (Ki67, CDK2, and PCNA) as a measure of proliferative index (Figure S5I). Using this index, we quantitatively compared proliferating vs. non-proliferating areas compared with areas with and without REC/CD34 proximity. In two AML patients examined, we found no significant difference in distribution in locations of proliferation (p = 0.27, Fisher’s Exact Test), suggesting proximity of RECs to primitive leukemic cells in patients is independent of proliferative status of these cell types. As this analysis is limited to area and transcript, we further investigated potential co-localization of RECs and CD34^+^ cells at the single-cell protein level in PDX mice. To achieve this, we applied multiplexed ion-beam imaging by time of flight (MIBI-TOF)^28^ technology to BM tissue sections of AML vs. CB xenografted mice (Figure 5F). RECs represented by CD74^+^CD68^+^ cells were found to be in closer proximity to CD34^+^ cells in the AML xenograft as compared with the CB xenografts (Figure 5G).

While it is historically the most robust surrogate marker of LSCs, using CD34 in this analysis has its shortcomings, as CD34^−^ LSCs exist and CD34^+^ cells that are not LSCs have been identified. However, to maintain consistency between conditions preserving comparative values in this analysis, we used the same markers for LSCs and healthy HSCs mainly due to an absence of precise markers that effectively discriminate LSCs from HSCs. Nonetheless, these observations collectively suggest leukemia-specific proximity of CD74^+^CD68^+^ cells and CD34^+^ primitive cells, and a potential support role for RECs in AML regeneration that forms the basis of functional testing.

### RECs catalyze leukemic regeneration by supporting LSCs

**Figure 6:**
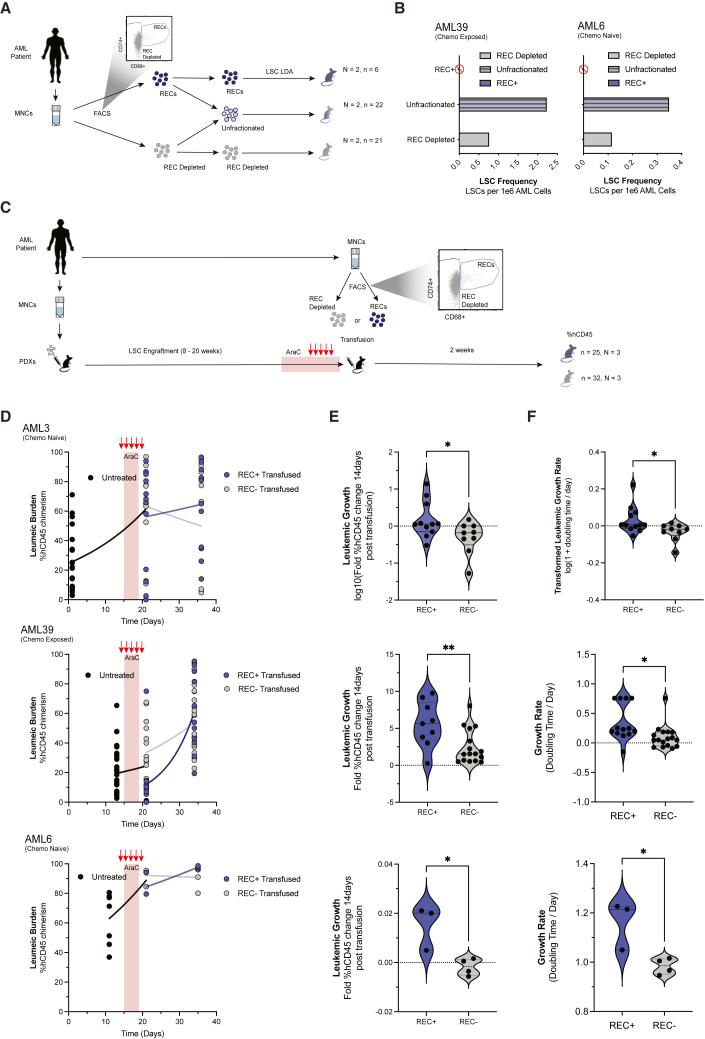
RECs catalyze leukemic regeneration by supporting LSCs (A) Experimental visual of REC loss of function limiting dilution analysis experiment. (B) Bar graph of estimated AML LSC frequency when RECs are depleted, present, and isolated (N = 2). (C) Experimental visual of REC gain-of-function transfusion experiment. (D) Growth of AML grafts before, during, and after REC or control transfusion (N = 3) AML growth is greater in PDXs that received REC transfusions as compared with control conditions calculated by (E) change in chimerism from transfusion time point to readout time point (13–16 days) and by (F) growth rate (doubling time per day) calculated by exponential growth fits (N = 3, Student’s t test, ∗∗p < 0.01, ∗p < 0.05). See also Figure S6, Table S1.

To functionally examine the properties of RECs, we assayed leukemic activity in both cellular loss and gain-of-function experiments. RECs were depleted from AML samples via FACS (representative FACS plots Figures S6A–S6C) and limiting dilution transplants (LDAs) were performed on the REC-depleted AML samples as compared with the unfractionated samples to obtain LSC frequency with and without RECs present (N = 2, Figure 6A). Notably, this experiment was designed by creating the unfractionated population by re-combining sorted RECs and REC-purified populations to their initial proportions to avoid any biases introduced by FACS. Although RECs are devoid of any intrinsic LSC capacity, REC-depleted AML samples contained less functional LSCs (Figure 6B), suggesting removal of RECs reduced supportive function to LSCs for survival and disease regeneration *in vivo*.

In cellular gain-of-function experimentation, the proportion of RECs present during leukemic regeneration was increased by transfusion of excess RECs. Between 50 and 80k FACS-purified patient-matched RECs were intrafemorally transfused (or cell number matched REC-depleted cell control) into patient-matched AraC-treated PDXs 2 days post chemo exposure (N = 3, Figure 6C, representative FACS plots Figures S6A–S6C). As RECs are non-self-sustaining, we postulated a transient increase of hCD45% following transfusion and traced the leukemic graft growth for the next 14 days (Figure 6D) by comparing the hCD45% chimerism before intrafemoral transfusion and 14 days later. REC-transfused grafts had a greater change in engraftment levels compared with REC-depleted transfused grafts (Figure 6E), and a greater growth rate calculated by the doubling time of the exponential curve fit (Figure 6F). This increase in AML regeneration in the presence of additional RECs demonstrates RECs augment leukemic growth following chemotherapy exposure. To determine whether the increase in chimerism was cell-extrinsic in nature, RECs were transfused into non-engrafted AraC-treated mice. No hCD45% chimerism was detected in these mice (Figure S6D) and therefore any growth from REC transfusion is non-cell autonomous. Notably, these experiments were performed using AML patients exposed to chemotherapy vs. untreated patients, with similar effects of augmenting AML regeneration with addition of RECs. Accordingly, although derived through enrichment following chemotherapy exposure in PDX models, these results indicate RECs play an LSC support cell function independent of chemotherapy exposure.

Overall these findings reveal RECs have prognostic and clinical utility beyond long-term survival outcomes alone, including revealing new and more precise targets that are not restricted to various LSC populations transcriptionally profiled previously.^29^^,^^30^^,^^31^

## Discussion

Our study identifies an accessory population of leukemic patient origin that acts like a catalyst for AML regeneration post chemotherapy. RECs have functional and prognostic value and may be due to the original approach used to identify RECs in our study that examine acute responses to regeneration following chemotherapy. These dynamics, originally defined in PDX models of AML regeneration, would be masked when performing molecular analysis on static patient samples obtained at defined stages of therapeutic management, e.g., Dx, CR, and relapse. Functional and molecular characterization of RECs is consistent with a non-autonomous role of leukemic CD74^+^CD68^+^ RECs for stem cell-driven AML regeneration. This adds to the mounting evidence for non-canonical cellular mechanisms of leukemic regeneration and disease relapse in response to therapy. The biological and clinical relevancy of a non-primitive gene score for RECs departs but collaborates with conventional canonical viewpoints of LSC-based AML regeneration. Our findings contribute to the foundational work from several studies identifying non-stem/progenitor gene expression properties that correlate to leukemic chemo-resilience and regeneration,^11^^,^^12^^,^^13^ and that more differentiated leukemias can have worse overall outcomes and treatment response to venetoclax,^32^^,^^33^ but these studies remain disconnected. As RECs are enriched for gene expression patterns identified in these separate reports, including cellular senescence, inflammation, oxidative phosphorylation (Figure S2K), and the leukemic regenerating cell gene score, RECs provide a basis for consolidation of these previously unlinked observations in AML.^11^^,^^12^^,^^13^ Furthermore, RECs allow an immunophenotypically definable cellular entity to be investigated and utilized, thus extending beyond the limited use of gene expression profiling. As sources of gene expression upregulation may be ambiguous and could be due to cellular response to chemotherapy exposure and not innate chemo-resistance, functional demonstration of immunophenotypically purified REC regeneration augmentation (Figure 6) leaves less ambiguity for future study and REC translational impact. To our knowledge, this is the first AML single-cell transcriptomics analysis that has resulted in the identification of an immunophenotypically defined cell involved in disease regeneration, as well as the initial demonstration of a role for non-stem/progenitor cells of leukemic origin to contribute to AML disease.

Our findings are based on our study’s approach that incorporates multiple post-chemotherapy time points and paired functional stem cell biology assays necessary to define the unique transcriptomic profile of transiently cytoreduced leukemia and leukemic graft regeneration. AML regeneration post chemotherapy allowed temporal tracking of both disease burden and progenitor activity during leukemic graft recovery to obtain a non-patient-specific gene score predictive of OS, EFS, and treatment response in independent patient cohorts. Notably, neither transcriptional REC clusters, nor their derived gene scores, exhibit similarity to primitive gene expression. As such, RECs serve a cellular therapeutic target with a defined phenotype that possesses prognostic value to AML patients as a biomarker of therapy response and long-term survival. Our results uncover a principle in AML disease suggesting cancer stem cell-driven regeneration is not solely cell autonomous in nature and involves other complex cellular elements exemplified by CD74^+^CD68^+^ RECs. This emphasizes the importance of cell-extrinsic factors that support chemotherapy resilience in stem cell-driven relapsed disease that may be applicable to other tumors sustained by cancer stem cells.^13^^,^^34^ RECs may represent a cellular bridge to the microenvironment for LSCs as RECs are products of LSC differentiation to sustain leukemia, and act as a survival response to chemotherapy injury that target LSCs. As such, the incorporation of REC detection into measurable residual disease (MRD)^35^ approaches could aid in the precision and fidelity of MRD measurement by including REC phenotype frequency alone or in combination with AML-patient-specific DNA mutations and existing MRD phenotype,^35^ as opposed to binary absence or presence of mutation detection currently used. This would require future multi-site clinical trials temporally following individual patients over months/years for further validation and is part of the ongoing goals of our group. Proximity of RECs to primitive leukemic cells requires further study to determine if direct contact interactions or paracrine signaling is involved in the supportive role of RECs to leukemic regeneration. Furthermore, as spatial transcriptomics may have value in disease treatment management using AML patient biopsies, larger cohorts of patients with this analysis may provide an approach to determine the prognostic value of REC proximity to leukemic subsets.

CD74 has been associated with cancer progression,^36^^,^^37^ proinflammatory immune response,^38^ has been implicated in regenerating tissues,^39^ and is considered a monocytic/macrophagic marker within the myeloid branch of the hematopoietic system. Along with other cell types, differentiated cells of the hematopoietic system including macrophages and monocytes help create the BM niche in a healthy hematopoietic system,^34^ supporting HSC survival and regulation. With an abundant role in tissue repair,^39^ the expression of CD74 in leukemic regeneration is consistent with the working theory that cancers are an over-healing wound,^40^ and suggest leukemic transformation hijacks healthy repair mechanisms for malignant growth and support by generating its own supportive cells. CD68 is a marker of macrophages and has been implicated with tumor-associated macrophages (TAMs) in AML.^41^ Although perhaps providing a similar role, the leukemic origin of RECs (Figure 5C) separate our findings from TAMs in AML disease. RECs are a product of leukemic transformation that require altered states of differentiation from normal hematopoiesis and are needed to survive aggressive chemotherapy injury. This may signify a change in the BM niche during leukemogenesis that preferentially supports LSCs over HSCs.^34^ We propose that LSC-generated monocyte and macrophage-like blast cells likely support leukemic regeneration, perhaps through a leukemic BM niche-like support system that remains to be understood. Nonetheless, directed therapies toward leukemic-derived cells such as RECs via drug targeting, antibody-based inhibition, or immunotherapy using CAR engineering would provide a first-in-class approach for combating AML relapse and warrants further investigation by the biomedical community.

### Limitations of the study

For PDX modeling, we have used the NSG mouse model, in which only a fraction of AML samples engraft. Often, the samples that engraft in NSGs are from patients who have more aggressive disease. Therefore, there is an implicit skew toward aggressive AML in the patient samples used for the REC identification via scRNA-seq, and GOF LOF functional validation of RECs. It is important to note that although functionally characterized, further in-depth genetic or clonal analysis of RECs in AML patients is needed. There is a possibility the RECs define, or are part of, a distinct molecular subgroup of AML patients, which has yet to be properly investigated. Furthermore, it is possible that other cells with different molecular phenotypes also contribute to non-canonical regeneration outside of RECs, and/or RECs are currently a heterogeneous population the requires additional fractionation for various supportive roles of LSC-driven regeneration. Despite these caveats, RECs retain predictive capacity by gene expression (Regen71) and immunophenotype (CD74/CD68), suggesting RECs play a significant role in a large fraction of AML cases but may be increasingly powerful should additional stratification of AML patients be applied. Future studies will require biological process of non-canonical regeneration to be investigated to ultimately reveal exactly how RECs support and potentially interact with primitive AML stem cell/progenitors that has not been precisely defined in our current study.

## STAR★Methods

### Key resources table

**Table:** 

REAGENT or RESOURCE	SOURCE	IDENTIFIER
**Antibodies**		

PE-Cy7 mouse anti-human CD68	BD Biosciences	Cat#585595; RRID:AB_2739298
FITC mouse anti-human CD163	BD Biosciences	Cat# 563697, RRID:AB_2738379
PE mouse anti-human CD13	BD Biosciences	Cat# 347837, RRID:AB_400355
V450 mouse anti-human CD44	BD Horizon	Cat#561292; RRID:AB_10612016
FITC mouse anti-human CD45	BD Biosciences	Cat#347463; RRID:AB_400306
V450 mouse anti-human CD45	BD Biosciences	Cat#642275; RRID:AB_1645755
APC mouse anti-human CD33	BD Biosciences	Cat#551378; RRID:AB_398502
PE mouse anti-human CD33	BD Biosciences	Cat#347787; RRID:AB_400350
APC mouse anti-human CD34	BD Biosciences	Cat#555824: RRID:AB_398614
PE mouse anti-human CD34	BD Biosciences	Cat#555822; RRID:AB_396151
FITC mouse anti-human CD19	BD Biosciences	Cat#555412; RRID:AB_395812
APC mouse anti-human CD3	BD Biosciences	Cat#555335 RRID:AB_398591
APC mouse anti-human CD117	BD Biosciences	Cat#341096; RRID:AB_400563
PE mouse anti-human CD74	BioLegend	Cat#326808; RRID:AB_2075505
APC mouse anti-human CD16	BD Biosciences	Cat#561304; RRID:AB_10714780

**Biological samples**		

Primary AML patient samples	Juravinksi Hospital and Cancer Center London Health Sciences Center	N/A
AML patient-derived xenografts	Juravinksi Hospital and Cancer Center London Health Sciences Center	N/A
Healthy human donor hematopoietic samples	Labor and Delivery Clinic at McMaster Children’s Hospital; Juravinksi Hospital and Cancer Center	N/A
Healthy human blood donor-derived xenografts	Labor and Delivery Clinic at McMaster Children’s Hospital; Juravinksi Hospital and Cancer Center	N/A
AML and Healthy BM biopsy tissue sections	London Health Sciences Center	N/A

**Chemicals, peptides, and recombinant proteins**		

7AAD	Backman Coulter	A07704
Trypan Blue	ThermoFisher Scientific	T10282
Cytosine β-D-arabinofuranoside	Sigma-Aldrich	C1768-1G
PBS	Wisent Bioproducts	Cat#311-425-CL
IMDM	Gibco	Cat#12440-063
EDTA	ThermoFisher - Invitrogen	AM9260G
RNase zap	Invitrogen	Cat#AM9780

**Critical commercial assays**		

Methocult	STEMCELL Tech.	Cat#H4434
DNeasy blood and tissue kit	Qiagen	Cat#69506
EasySep Human CD3 Positive Selection Kit	STEMCELL Tech.	Cat#17851
Single Cell 3′ Library Kit v3	10X Genomics	Part No: 1000078
Single Cell 3′ GEM Kit v3	10X Genomics	Part No: 1000094
3′ Feature Barcode Kit	10X Genomics	Part No: 1000262
Spatial Transcriptomics	10X Genomics	Part No: 1000185
ddPCR™ Supermix for Probes	BioRad Laboratories	Cat#1863010

**Deposited data**		

scRNA seq	This Paper	GEO Accession: GSE255245
Spatial Transcriptomics	This Paper	GEO Accession: GSE255217

**Experimental models: Organisms/strains**		

NOD.CB17-Prkdc^scid^/J (NOD/SCID)	The Jackson laboratory	RRID:IMSR_JAX:001303
NOD.Cg-Prkdc^scid^Il2rg^tm1wjl^/SzJ (NSG)	The Jackson laboratory	RRID:IMSR_ARC:NSG JAX:05557

**Software and algorithms**		

FACSDiva	BD	http://www.bdbiosciences.com/us/instruments/research/software/flow-cytometry-acquisition
CytExpert	Beckman Coulter	https://www.beckman.com/flow-cytometry/research-flow-cytometers/cytoflex/software
FlowJo 10.8	FlowJo, LLC	https://www.flowjo.com
Prism version 7.0	GraphPad	https://graphpad.com
R 4.0.3	R Foundation	https://www.r-project.org/
Seurat 4.0.2	Sajita Lab	https://satijalab.org/seurat/
Cell Ranger 6.0.2	10X Genomics	https://support.10xgenomics.com/single-cell-gene-expression/software/pipelines/latest/what-is-cell-ranger
Space Ranger 1.3.1	10X Genomics	https://support.10xgenomics.com/spatial-gene-expression/software/pipelines/latest/what-is-space-ranger
Loupe Browser 6.0.0	10X Genomics	https://www.10xgenomics.com/products/loupe-browser/downloads
Celldex 1.0.0	Bhattacharya Lab	https://bioconductor.riken.jp/packages/3.12/data/experiment/html/celldex.html
GSEA 4.2.3	Broad Institute	https://www.gsea-msigdb.org/gsea/index.jsp
QuantaSoft Analysis Pro software v1.0.596	Bio-Rad Laboratories	QuantaSoft Analysis Pro software v1.0.596

### Resource availability

#### Lead contact

Further information and requests for resources and reagents should be directed to and will be fulfilled by the corresponding author, Mickie Bhatia (mbhatia@mcmaster.ca).

#### Materials availability

This study did not generate new unique reagents.

#### Data and code availability

Single-cell RNA-seq and spatial transcriptomics data have been deposited at GEO and are publicly available as of the date of publication. Accession numbers are listed in the key resources table. This paper analyzes existing, publicly available data. These accession numbers for the datasets are listed in the key resources table. Processed scRNA seq data reported in this paper will be shared by the lead contact upon request. All original code has been deposited and will be publicly available by date of publication. DOIs are listed in the key resources table. TARGET AML and TCGA L-AML RNA sequencing and clinical data can be found at the NIH National Cancer Institute GDC Data Portal: https://portal.gdc.cancer.gov/. AFFYMETRIX and survival data of the 79 patient cohort of CN AML patients^23^ can be accessed through the GEO: GSE12417 accession. Any additional information required to reanalyze the data reported in this work paper is available from the lead contact upon request.

### Experimental model and subject details

#### Primary human samples

Healthy human hematopoietic cells were isolated from BM and mobilized peripheral blood of adult donors or from umbilical cord blood. Primary AML specimens were obtained from peripheral blood apheresis or BM aspirates of consenting AML patients. AML samples and adult sources of healthy hematopoietic tissue were provided by Juravinski Hospital and Cancer Center and London Health Sciences Center (University of Western Ontario). The Labor and Delivery Clinic at the McMaster Children’s Hospital provided healthy cord blood samples. All samples were obtained from informed consenting donors in accordance with approved protocols by the Research Ethics Board at McMaster University and the London Health Sciences Center, University of Western Ontario. Details of AML patient samples are outlined in Table S1. Mononuclear cells (MNCs) were recovered by density gradient centrifugation (Ficoll-Paque Premium; GE Healthcare) followed by red blood cell lysis using ammonium chloride solution (Stemcell Technologies). Lineage depletion of healthy hematopoietic samples was carried out using EasySep immunomagnetic cell separation (Stemcell Technologies), according to the manufacturer’s instructions. Flow cytometry analyses involving treatment success (Figure 4A) are of samples retrieved at onset of AML diagnosis, and patients subsequently treated with standard induction chemotherapy regimens consisting of 7-day infusions of cytarabine (100 mg/m^2^) plus daunorubicin on days 1–3 (60 mg/m^2^). To minimize variability, patients were selected for this analysis that had not undergone hematopoietic stem cell transplants.

Trephine BM biopsies (1.5–2 cm) were obtained from consenting AML patients enrolled under a bone marrow transplantation study (The Ottawa Hospital, Ontario, Canada) and from consenting healthy donors undergoing BM assessment for stem cell transplants (London Health Sciences Center, University of Western Ontario) under the approval of the research ethics office of the Ottawa Health Science Network Research Ethics Board (OHSN-REB, 20130729-01H) and London Health Sciences Center, University of Western Ontario (IRB#00000940). Following collection of trephine biopsy, sample was stored in 10% neutral buffered formalin (NBF) for a maximum of 24 h. The biopsy was washed 3 times in 1X PBS for a duration of 5 min per wash (Wisent Inc., Cat#311-010-CL) before proceeding to embedding in paraffin according to standard histology procedures.

#### Murine recipients and xenograft assays

Mice were bred and maintained at the McMaster Stem Cell and Cancer animal barrier facility. All experimental procedures were approved by the Animal Council of McMaster University. NOD/SCID or NSG mice were used as xenograft recipients, and xenotransplantation was performed as previously described.^13^ Briefly, 6–10 week old recipient mice were sublethally irradiated (315–325 Rads, using a 137Cs g-irradiator) 24 h prior to intravenous transplantation of primary human samples. When NSG recipients were used, primary human samples were CD3 depleted before injection. Both male and female mice were used, however sex (defined by chromosomal genotype) was controlled within individual experiments. For limiting dilution analysis experiments, the highest dose of injections was determined by using sample specific cell doses that had been previously known to engraft, and the lowest doses were at least 20x less concentrated. Within an experiment, at least 4 doses were used per condition with at least two mice per condition/cell dose combination. Within each experiment, each engrafting condition was injected into the same number of mice at the same doses to minimize variability. In all xenograft experiments, 6–18 weeks following transplantation, BM cells were recovered by mechanical dissociation and analyzed by flow cytometry. BM cellularity was quantified using trypan blue exclusion. To evaluate human cells of xenografts in downstream analyses such as functional progenitor content and scRNA seq, xenografted human cells were purified by fluorescence-activated cell sorting (FACS) on CD45^+^ and CD33^+^ co-expression, or by mouse cell exclusion using magnetic cell isolation (mouse CD45 and mouse Ter119; Miltenyi Biotec) and subsequently seeded in methylcellulose (STEMCELL Technologies H4434) or directly into the described 10X Genomics scRNA sequencing protocol. In experiments where residual human AML cells were isolated for methylcellulose progenitor assays, cell seeding numbers were based on the total number cells recovered as well as the known requirements for cell number input for the respective assays (characterized independently for different AML patient samples). Longitudinal *in vivo* monitoring of human leukemic chimerism was carried out by serial BM aspiration. 5-10μl of BM cells were collected from femurs of anesthetized recipient mice; when repeated, the procedure alternated femurs. For *in vivo* therapy testing, mice were treated with AraC (Sigma-Aldrich) once human grafts were established (5–18 weeks post-transplant). 50 mg/kg AraC was delivered daily by subcutaneous injections over five consecutive days at doses optimized by both our group and others.^11^^,^^13^ Bi-daily weight measurements were used to ensure that an appropriate dose per weight ratio was sustained throughout each treatment. Mice were allocated to treatment groups based on pre-treatment BM aspirates, to ensure similar starting levels of human chimerism across groups. If no initial assessment of chimerism was performed, mice were randomly allocated to experimental groups, assuring that cage mates were distributed across different groups. When utilized, cell transfusions (Figure 6C) were administered intrafemorally, two days following five-day AraC treatment and immediately following BM aspirations from the same femur. To minimize the number of procedures, 2 days post-AraC hCD45^+^ readings were taken immediately prior (during the same procedure) to the cell transfusion. Due to this, when allocating mice to transfusion treatment groups, hCD45^+^ chimerism information was unavailable. Therefore, to limit variability across groups during these experiments, we used pre-chemotherapy hCD45^+^ percentages and split groups within cages. To assess results of transfusion experiments, BM was isolated via BM aspiration or mechanical dissociation of the non-injected femur 13–16 days (conditions within experiments were time matched) following intrafemoral transfusion. Cell number was matched per experiment, injecting between 50 and 90k cells (REC^+^ or REC depleted matched in each experiment) per mouse in 30uL PBS (0.01% FBS).

### Method details

#### Droplet based scRNA sequencing

3′ scRNA sequencing experiments were performed directly on cells purified by the FACSAria II: hCD45+ CD33^+^ 7AAD-when from xenograft source; 7AAD-when directly from patient tissue. During all preparation steps, cells were kept at 2-6°C. Manufactures recommendation’s (User Guide, CG000388) were following to create single cell libraries. When cell multiplexing was utilized (PN#1000262), up to three samples were multiplexed into a single run, and samples that were multiplexed together were always biological replicates. Gene expression and cell multiplexing libraries were sequenced on the NovaSeq SP flow cell (TCAG, SickKids) using the recommended parameters.

#### Spatial transcriptomics

Prior to performing spatial transcriptomics experiment, DV200 assessment was done on all the samples with a minimum criterion set at 30%. RNA was extracted from paraffin curls (4 curls at 10 μm per tissue) using Qiagen’s RNeasy Mini Kit (Cat#74104). All working space and instruments were treated with RNase zap (Invitrogen, Cat#AM9780) prior to RNA extraction, and gentle scoring was performed around the tissue to minimize excess paraffin during curl collection. Spatial transcriptomics experiment was performed using Visium Spatial Transcriptomics (10X genomics, Cat#1000185), following materials and methods according to the manufacturer’s website (User Guide, CG000407). Gene expression libraries were sequenced on the NovaSeq SP flow cell (TCAG, SickKids) using the recommended parameters. Analysis and visualizations were performed by manufacturer provided Loupe Browser. Co-localization of putative LSCs to RECs was determined by areas of CD34^+^ expression AND areas that of CD74^+^/CD68^+^ expression (Expression >0 reads).

#### Bioinformatics pipelines

ScRNA sequencing reads were counted and aligned using Cell Ranger software provided by 10X Genomics. Individual scRNA sequencing datasets were integrated, and batch corrected using Seurat’s integration protocol, and QCed using standard exclusion metrics (%mitochondrial genes >3∗SD + median_sample_, (cell features < median_sample_ – (3∗SD)). An integration anchor of healthy BM was used when samples of different patient backgrounds were being merged to minimize variation on interpatient heterogeneity. The K nearest neighbor clustering methodology was used to identify clusters in datasets described. Clusters were excluded from analyses on a per sample basis if the percent of the cluster within the sample of the integrated dataset was less than the smallest cluster of the sample dataset when not integrated with other data. We considered these clusters bioinformatic products of the integration protocol, called them non-substantive clusters, and thus were excluded from downstream calculations. Visuals were created using the Seurat’s R package built-in visualization program. We followed manufacturers recommendations when utilizing the celldex package for assigning cell types using data from the human primary cell atlas. We followed manufacturers recommendations when utilizing the Seurat package for assigning cell cycle phase. The Seurat Package’s FindAllMarkers function was used to to perform Wilcoxon rank-sum test to derive DEG (defined by Log2FC > 0.25, padj >0.01). Sequencing reads from spatial transcriptomics protocols were counted, aligned, and aggregated using manufacturer provided Space Ranger and Loupe Browser, following pipeline provided by the manufacturer. Visualizations of these datasets were created using Loupe Browser and GraphPad Prism. Areas were considered positive for a transcript if expression was detected, and considered negative if no expression was detected.

#### Fluorescence-activated cell sorting and flow cytometry

Immunophenotyping for human hematopoietic cell surface markers was carried out using the following antibodies: V450-conjugated anti-CD45 (1:100; 2D1), APC-conjugated anti-CD33 (1:300; WM-33), PE-conjugated or APC-conjugated anti-CD34 (1:200; 581), FITC-conjugated anti-CD19 (1:100; HIB19) and conjugated anti-CD117 (1:200). To evaluate candidates from our REC gene signature at the protein level, we identified gene targets with available commercial antibodies that had been validated for flow cytometry. CD74 CD68, CD14, CD44, CD163. 7-aminoactinomycin D (7AAD, Beckman Coulter) exclusion was used to discriminate live cells and was always used during cell sorting processes. When appropriate, fluorescence minus one control were used to optimize gating strategies for target cell populations. For scRNA sequencing experiments directly from patient tissue, viable MNCs were purified based on side scatter and forward scatter gating and 7AAD exclusion. To FACS isolate cells from PDX models for downstream applications of scRNA sequencing and CFU assays, MNCs from human grafts were isolated by using forward scatter and side scatter gates, 7AAD exclusion, and hCD45+ CD33^+^ gates. RECs were purified from primary AML samples using forward scatter and side scatter gates, 7AAD exclusion, and CD74^+^ CD68^+^ gates. Post-sort purities were routinely >95%. FACS sorting was performed using a FACSAria II sorter, and flow cytometry analysis was performed with an LSRII Cytometer (BD), or CytoFlex LX (Backman Coulter). FACSDiva (BD) and CytExpert were used for data acquisition, and FlowJo software (Tree Star) was used for analysis.

#### MIBI-TOF processing and analysis

We sectioned tissues 4μm in thickness onto gold coated MIBIslides. The slides baked at 65°C for 1 h, followed by deparaffinization and rehydration in sequential washes in xylene (3x), 100% ethanol (3x), 95% ethanol (2x), 70% ethanol (2x), and MIBI-water. Antigen-retrieval in a pH 9 Target Antigen Retrieval Solution (DAKO Agilent) occurred at 125°C for 40 min in a Decloaking Chamber (BioCare Medical). After cooling to room temperature, we washed the slides twice in TBS-T (IonPath). We incubated the tissue in a blocking buffer consisting of 3% normal donkey serum (Jackson ImmunoResearch) in TBS-T for 20 min. We incubated the slides with blocking-buffer-diluted antibody panel consisting of metal-tagged antibodies supplied by IonPath overnight at 4°C in a moisture chamber. After overnight incubation, we fixed the tissues and dehydrated in sequential washes in TBS-T (3x), 2% glutaraldehyde (5min), Tris pH 8.5 (3x), MIBI-water (2x), 70% ethanol (2x), 90% ethanol (2x), 95x ethanol (2x), 100% ethanol (3x). We stored the slides in a desiccator prior to MIBIscope analysis. We collected spectral images of mouse femurs using an IonPath MIBIscope with Multiplexed Ion-Beam Imaging technology. Xenon primary ions from a Hyperion ion gun rastered across the slide to sputter stained tissue into a plume of secondary ions detected by mass spectrometry by time-of-flight to reconstruct the spectral images, on a pixel-by-pixel basis, of each channel consisting of a single stained antibody. A more detailed description of the Multiplexed Ion-Beam Imaging technology appears in Keren et al. (2018).^42^ With the assistance of a pathologist, 400 × 400μm fields of view (FOVs) inside the lesions. Multiplexed raw image sets were denoised and aggregate filtered using IonPath’s MIBI/O and the default correction settings. These processed image TIFF files represented the dataset. We performed nuclei segmentation with the input of the nuclear-stained and the membrane-stained marker channels using Mesmer^43^ and we stored the segmentation mask images as TIFF files for further analysis in MATLAB and R Studio. We extracted single-cell data for all cell objects defined by the segmentation masks using a custom R script and packages as previously described.^44^^,^^45^^,^^46^ We asinh-transformed with a cofactor of 1. To classify cell types based on their marker expression levels, we used the Bioconductor 'FlowSOM' R package.^47^ The algorithm clustered the 534,012 total cells from the cohort into 100 FlowSOM clusters. By inspecting a heatmap displaying normalized individual marker intensities, we annotated each of the 100 clusters into 16 meta-clusters, which included signatures that represented CD34^+^ cells and CD74^+^CD68^+^ cells for downstream proximity analysis. We used the CytoMAP software^48^ to perform single-cell spatial analysis with the aim to determine the proximity of CD74^+^CD68^+^ cells to all CD34^+^ cells present in the FOVs. The algorithm achieved this by using the cell types and their positions in the image to calculate the distance between all CD34^+^ cells and the nearest cell for CD74^+^CD68^+^ cells.

#### Droplet Digital polymerase chain reaction

Detection of NPM1 c.863_864insTCTG (COSMIC 17559), TP53c842A-T, and idh2 c515 was performed on the QX200 Droplet Digital PCR system (Bio-Rad Laboratories, Inc., Hercules, CA, USA) using TaqMan(tm) Liquid Biopsy dPCR Assay Hs000000064_rm (Life Technologies, Carlsbad, CA, USA). The 20 mL reaction mix consisted of 10 mL of 2x ddPCR SuperMix for Probes (Bio-Rad Laboratories), 0.5 mL of the 40X assay, 9.5 mL water and 1 mL of 30–50 ng/mL genomic DNA. The assay was tested by temperature gradient to ensure optimal separation of reference and variant signals. Cycling conditions for the reaction were 95C for 10 min, followed by 45 cycles of 94C for 30s and 60C for 1 min, 98C for 10 min and finally a 4C hold on a Life Technologies Veriti thermal cycler. Data were analyzed using QuantaSoft Analysis Pro software v1.0.596 (Bio-Rad Laboratories).

#### Progenitor frequency assays

The clonogenic capacity of leukemic progenitors was evaluated by colony-forming unit (CFU) assays. Briefly, AML cells (500–25000 cells/well) were seeded in semisolid methylcellulose media (Methocult #H4434; Stemcell Technologies) according to established protocols. Progenitor assays of xenografted leukemic cells were performed following human cell purification as described above. Individual CFU wells were seeded from multiple mice.

#### Multivariate survival analysis

TARGET AML and TCGA L-AML RNA sequencing and clinical data were accessed from the NIH National Cancer Institute GDC Data Portal: https://portal.gdc.cancer.gov/. Cohort sizes were adjusted due to the gene expression and survival (OS and EFS) data readily accessible through the GDC Data Portal. When filtering the TARGET AML patient cohort to achieve a cohort of patients with consistent induction treatment, we used the Gemtuzumab ozogamicin treatment tab and selected patients with no Gemtuzumab ozogamicin treatment from the linked AAML0513^49^ and AAML03P1^50^ trials. These patients received ADE10 (cytarabine 100 mg/m^2^/dose (3.3 mg/kg) every 12 h on Days 1–10; dau-norubicin 50 mg/m^2^/dose once daily (1.67 mg/kg) on Days 1,3, and 5; and etoposide 100 mg/m^2^/dose (3.3 mg/kg) oncedaily on Days 1–5) without the use of Gemtuzumab ozogamicin.^49^ Gene scores were calculated as per quantification and statistical analysis section. Missing clinical data were replaced by Bayesian polytomous regression or logistic regression. R v3.5.1 (using packages survival v3.2-7, survminer v0.4.8, and mice v3.11.0) were used for this analysis.

### Quantification and statistical analyses

Summarized data are represented as mean ± standard deviation. Statistical comparisons were analyzed using unpaired student’s t-tests (two-tailed), paired t-tests, one-way analysis of variance tests (ANOVAs) followed by Tukey’s multiple comparison tests, two-way ANOVAs, Fisher’s exact test., chi square test, or Mantel Cox tests Any deviations from normal distribution or homogeneity of variances were corrected by log_10_ transformation prior to parametric statistical tests, unless transformation did not resolve heterogeneity in variances in which non-parametric tests were applied. Datasets that had negative or zero values prior to transformation to reduce heterogeneity of variances were translated by +1 prior to log transformation. Prism software (version 7.0; GraphPad), R v3.5.1 (using packages survival v3.2-7, survminer v0.4.8, and mice v3.11.0) and MedCalc software (v20.110) was used for statistical analysis and p < 0.05 was considered statistically significant unless otherwise specified in the figure captions. Survival times were calculated from the date of sample collection, using previously established criteria for OS and EFS by subtracting the date of sample collection from the date of death (OS) or from the date of relapse (EFS).^51^ The expression score from every patient within a specific probe/gene was normalized to the mean expression of that probe to adjust to inter-probe transcriptional variance. To assign a value of a gene score to each patient, this value was calculated and averaged for each probe/gene of a select gene score. When dividing patients into high and low scores, the cohort was stratified into gene score high and low by arrangement around the gene score median. The Kaplan Meier method was used for univariate survival analyses and multivariate Cox regression was used to evaluate independent predictors of survival. For multivariate survival analyses, missing data were replaced by Bayesian polytomous regression or logistic regression. Patients with primary refractory disease were assigned an EFS of 0 days.

## Supplemental information

Document S1. Figures S1–S6 and Tables S1 and S2

Data S1. Curation of gene lists used and/or developed, related to Figures 2–5

Document S2. Article plus supplemental information
