# Supplementary material for: Identification of cells of leukemic stem cell origin with non-canonical regenerative properties
Source: Cell Rep Med. 2024 Apr 5;5(4):101485. doi: 10.1016/j.xcrm.2024.101485 (PMC11031376; doi:10.1016/j.xcrm.2024.101485)
Supplement: Document S1. Figures S1–S6 and Tables S1 and S2 [file mmc1.pdf]

Cell Reports Medicine, Volume *5*

Supplemental information

Identification of cells of leukemic stem cell origin with non-canonical regenerative properties

Cameron G. Hollands, Allison L. Boyd, Xueli Zhao, Jennifer C. Reid, Charisa Henly, Amro ElRafie, David Boylan, Emily Broder, Olivia Kalau, Paige Johnson, Alyssa Mark, Jamie McNicol, Anargyros Xenocostas, Tobias Berg, Ronan Foley, Michael Trus, Brian Leber, Alejandro Garcia-Horton, Clinton Campbell, and Mickie Bhatia

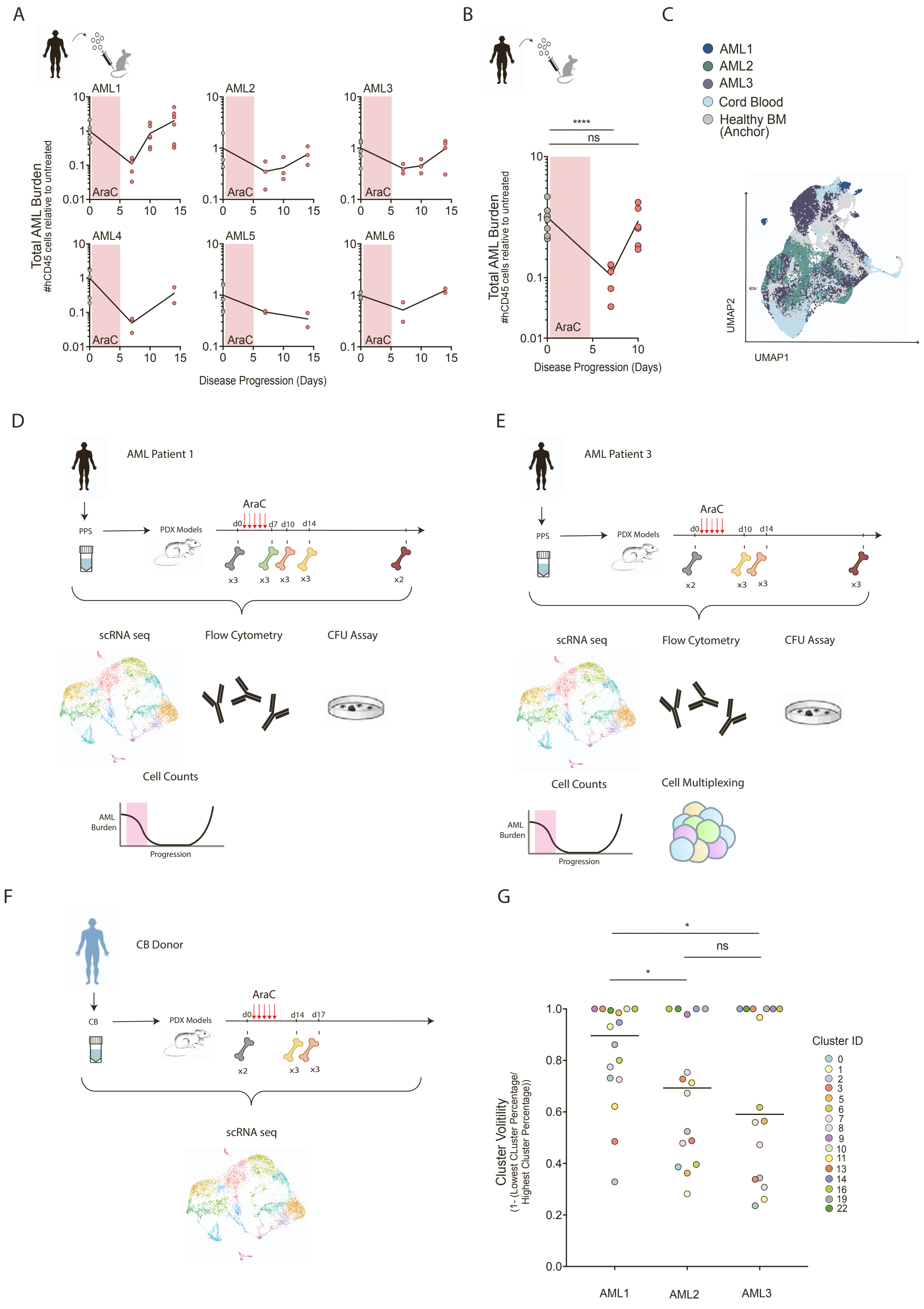

Figure S1: A battery of assays performed on AML PDXs following cytarabine treatment identifies distinct kinetics of AML response. Related to Figure 1. A. Total number of cells (hCD45Chimerism * Total Cells Harvested) in PDXs throughout a 5-day AraC treatment time course normalized to Untreated control from AML1-6 PDXs (N=6, n = 9-22 per AML). **B** Total AML burden of AML1 PDXs at Day 0, Day 7 and Day 10 timepoints of a 5-day AraC treatment normalized to Untreated (Day 0) (n = 5-7 per timepoint) **C** UMAP plot of all PDX cells in the analysis anchored by healthy BM, organized by tissue source (n = 78 500). **D-F.** Experimental outline for generating scRNA and paired analyses from AML patient 1, AML patient 3 and a CB donor. **G** A metric of cluster volatility (1 – [Lowest Cluster%/Highest Cluster%]) representing the proportional change of the cluster throughout leukemic regeneration of each shared and substantive cluster graphed by each AML (n = 16). Floating bar represents mean of each AML. ****p<0.0001, *p<0.05, ns p>0.05 by unpaired t tests (**B**), or paired t tests (**G**).

Table S1: AML patient/sample information and annotation. Related to Figures 1-6:

Sample ID	Clinical Stage	Tissue Source	Induction Therapy Details	Induction Treatment Success	Relapse	Time in remssion OR Time to relapse	PDX Reponder	Molecular/CG
1	AML Diagnosis	PB	7+3	Achieved CR	Y	150	Y	46,XX,FLT3 c. 1795_1818dup (70.1%); IDH2 419G>A (44.1%); NPM1 860_863dup (46.7%); DNMT3A 2645G>A (47.0%)
2	AML Diagnosis	PB	HDAC	Death before CR	N/A	N/A	N	Performed from cyropreserved DNA (GeneSeeq service): ERCC2: c.477+1G>A (51.21%); EZH2: c.382T>A(p.L128I) (98.45%); KRAS: c.34G>A(p.G12S) (50.06%); ETV6: c.331G>A(p.D111N) (50%); DNMT3A: c.2645G>A(p.R882H) (49.29%); MLLT3-KMT2A translocations
3	AML Diagnosis	Leukapheresis	N/A	Death before CR	N/A	N/A	N	46,XX,del(5)(q22q35)[cp3]/45~46,idem,del(7)(q32)[cp2]/44~46,idem,t(1;12(p13;p13),del(2)(p23)[cp2]/42~46,idem,del(3)(p22p24),der(3)inv(3)(p21q21)del(3)(q21),del(7)(q32),add(18)(q21),add(20)(p12)[cp13]/ 44~46,idem,del(1)(p22p32),del(3)(p22p24),del(4)(q21),del(7)(q22q36),del(9)(q22q32),add(12)(q24.1)[cp5], Karyotype was from August 28, not at the time of sample collection (this was before the dx of leukemia was made)
4	AML Diagnosis	BM	7+3	Achieved CR	N	2854	Y	FLT3-D835 inconclusive (<1) CBFß/MYH11 implies abnormality in chromosome 16. Otherwise, this patient has a normal copy number. Karyotype was 46, XY [2] however this was low quality, CKIT
5	AML Diagnosis	Leukapheresis	7+3	Persistent Disease	N/A	N/A	N	46,XX[20],Negative for PML/RARA translocation by FISH Normal karyotype according to correspondence from AX; BM exam on July 3, 2015, NPM1, FLT3-ITD
6	AML Diagnosis	Leukapheresis	7+3	Persistent Disease	N/A		N	negative for FLT3 D835Y and NPM1
7	AML Diagnosis	PB	7+3	Achieved CR	N	1115	N/A	46, XX [20], positive NPM1 and FLT3-ITD
8	AML Diagnosis	PB	7+3	Achieved CR	Y	2609	N/A	46,XY [20], NPM1 positive, FLT3 negative
9	AML Diagnosis	BM	7+3	Persistent Disease	N/A	N/A	N/A	46,XX [20], positive for NPM1 and FLT3-ITD
10	AML Diagnosis	PB	7+3	Achieved CR	Y	Unknown	N/A	47,XX,+11[20], IDH2:c.515G>A, p.(Arg172Lys) (49.1%). PTPN11:c.179G>T, p.(Gly60Val) (46.3%), positive for IDH2
11	AML Diagnosis	Trephine BM biopsies	N/A	N/A	N/A	N/A	N/A	Unknown
12	AML Relapse	Trephine BM biopsies	N/A	N/A	N/A	N/A	N/A	Unknown
13	AML Diagnosis	BM	7+3	Achieved CR	N	2013	N/A	46,XX,add(4)(q31),t(16;16)(p13;q22)[25], nuc ish(5'MYH11,3'MYH11)x2,(5'MYH11 sep 3'MYH11x1)[188/200] nuc ish(5'CBFB,3'CBFB)x2,(5'CBFB sep 3'CBFBx1)[192/200], negative for CKIT
14	AML Diagnosis	BM	7+3	Achieved CR	N	1896	N/A	46,XX,t(8;21)(q22;q22)[25], positive for CKIT
15	AML Diagnosis	BM	7+3	Achieved CR	N	4395	N/A	46,XX,inv(16)(p13q22)[25], nuc ish(CBFBx2)(5' CBFB sep 3' CBFBx1)[180/200]
16	AML Diagnosis	PB	7+3	Achieved CR	N	731	N/A	Unknown
17	AML Diagnosis	BM	7+3	Achieved CR	N	1826	N/A	Unknown
18	AML Diagnosis	Leukapheresis	7+3	Achieved CR	N	1426	N/A	46,XX[8], Normal FISH result nuc ish(PML,RARA)x2[200], positive for FLT3-ITD and NPM1
19	AML Diagnosis	BM	7+3	Achieved CR	N	2182	N/A	46,XY,t(8;21)(q22;q22)[25]
20	AML Diagnosis	BM	7+3	Achieved CR	Y	506	N/A	46,XY,inv(16)(p13.1q22)[25], positive for KIT
21	AML Diagnosis	PB	7+3	Achieved CR	Y	202	N/A	46,XX[11], Normal FISH result nuc ish(CBFBx2)[200]
22	AML Diagnosis	PB	7+3	Achieved CR	Y	78	N/A	47,XX,+8[4]/46,XX[4], trisomy 8, MLL-[200/200], positive for NPM1 and FLT3-ITD
23	AML Diagnosis	PB	7+3	Achieved CR	Y	85	N/A	46,XY,+?del(4)(p?15),-22[2].nuc ish(MLLx2)[188], MLL NOT DETECTED BY FISH
24	AML Diagnosis	BM	7+3	Achieved CR	Y	464	N/A	46,XY[20]
25	AML Diagnosis	BM	7+3	Achieved CR	Y	520	N/A	46,XY,t(9;11)(p22;q23)[9]/47,sl,+mar[2]/46,XY[3].nuc ish(MLLx2),(5'MLLsep3'MLLx1)[181/200],(MYH11,CBFB)x2[198]. , t(9;11) isolated or with other abnormalities, 11q23 (MLL) abnormalities, isolated or with other abnormalities, excluding t(9;11),
26	AML Diagnosis	PB	7+3	Achieved CR	Y	78	N/A	47,XX,+8[4]/46,XX[4], trisomy 8, MLL-[200/200], positive for NPM1 and FLT3-ITD
27	AML Diagnosis	BM	7+3	Achieved CR	Y	177	N/A	46,XY [3]
28	AML Diagnosis	BM	7+3	Achieved CR	Y	294	N/A	46,XX,inv(16)(p13q22)[8], nuc ish(CBFBx2)(5'CBFB sep 3'CBFBx1)[172/200]
29	AML Diagnosis	PB	7+3	Persistent Disease	N/A	N/A	N/A	46,XY,t(1;14)(q32;q32)[5]/46,XY,del(1)(q42)[3]/46,XY[17],Karyotype was initially normal karyotype but then acquired abnormalities over the course of therapy. Note that some samples were collected when karyotype was normal.
30	AML Diagnosis	BM	7+3	Persistent Disease	N/A	N/A	N/A	46,XY[24]
31	AML Diagnosis	BM	7+3	Persistent Disease	N/A	N/A	N/A	42~46,X,-Y,t(2;15)(q37;q22),dic(5;17)(q11;p11),+10,add(11)(p15),-17,-18,-21,-22,+2~4mar[cp19]/ 62<3n>,XYY,+1,t(2;15)(q37;q22),-3,-5,+13,-17,-17,-18,-19,-19,-21,-22[1]/ 80~83<4n>,XXYY,der(1)t(1;11)(q32;q13)x2,t(2;15)(q37;q22)x2,-3,-5,dic(5;17)(q11;p11),-7,-11,-16,-17,-17,-18,-20,-21,-21,+2mar[cp2]/46,XY[3]
32	AML Diagnosis	BM	7+3	Persistent Disease	N/A	N/A	N/A	46,XX[22], Genetic workup not done at time of sample collection. Later sample collected May 21, 2015 was 74.85% FLT3-ITD mutant, FLT3-TKD neg (extra information from Eri Iida clinical file review), positive for NPM1
33	AML Diagnosis	BM	7+3	Persistent Disease	N/A	N/A	N/A	46,XY[20], FLT3 and NPM1 measured prior to sample collection (at UHN prior to referral to LHSC) NEGATIVE
34	AML Diagnosis	PB	7+3	Persistent Disease	N/A	N/A	N/A	47,XY,+8[9]/46,XY[4], 10% (20/200)trisomy 8
35	AML Diagnosis	PB	7+3	Persistent Disease	N/A	N/A	N/A	del(5) (q22q33), -7
36	AML Diagnosis	BM	7+3	Persistent Disease	N/A	N/A	N/A	46,XY
37	AML Diagnosis	BM	7+3	Persistent Disease	N/A	N/A	N/A	46,XY [3]
38a	AML Diagnosis	PB	7+3	Persistent Disease	N/A	N/A	N/A	Unknown
38b	Persistent Disease	PB	7+3	N/A	N/A	N/A	N/A	Unknown
39	Relapsed AML	Leukapheresis	7+3	Unknown	N/A	N/A	N/A	Unknown

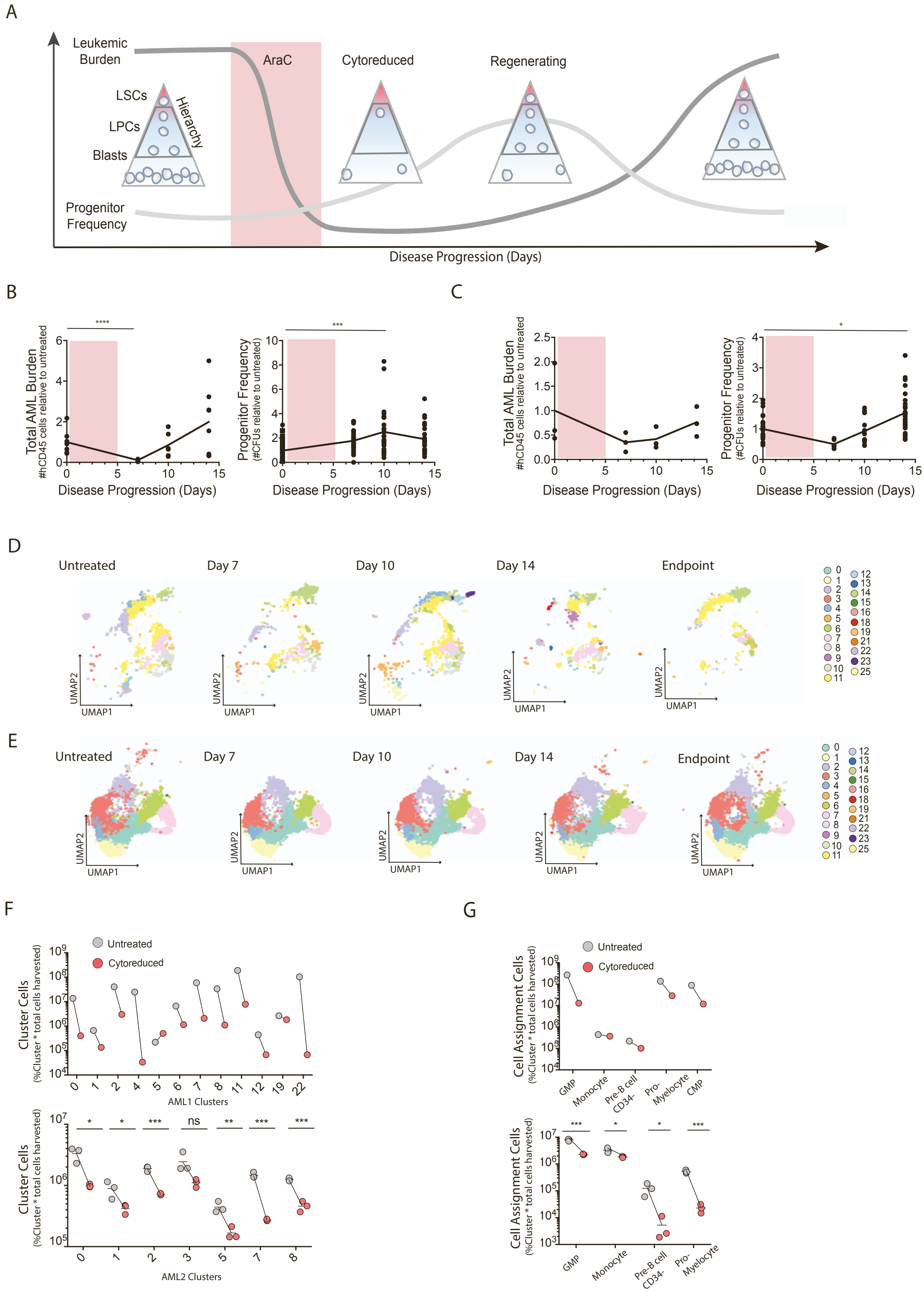

A
Leukemic Burden
AraC
Cytoreduced
Regenerating
LSCs
LPCs
Blasts
Hierarchy
Progenitor Frequency
Disease Progression (Days)
B
Total AML Burden
#hCD45 cells relative to untreated
Disease Progression (Days)
Progenitor Frequency
(#CFUs relative to untreated)
Disease Progression (Days)
C
Total AML Burden
#hCD45 cells relative to untreated
Disease Progression (Days)
Progenitor Frequency
(#CFUs relative to untreated)
Disease Progression (Days)
D
Untreated
Day 7
Day 10
Day 14
Endpoint
UMAP1
UMAP2
E
Untreated
Day 7
Day 10
Day 14
Endpoint
UMAP1
UMAP2
F
Untreated
Cytoreduced
Cluster Cells
(%Cluster * total cells harvested)
AML1 Clusters
AML2 Clusters
G
Untreated
Cytoreduced
Cell Assignment Cells
(%Cluster * total cells harvested)
GMP
Monocyte
Pre-B cell CD34-
Pro-Myelocyte
CMP

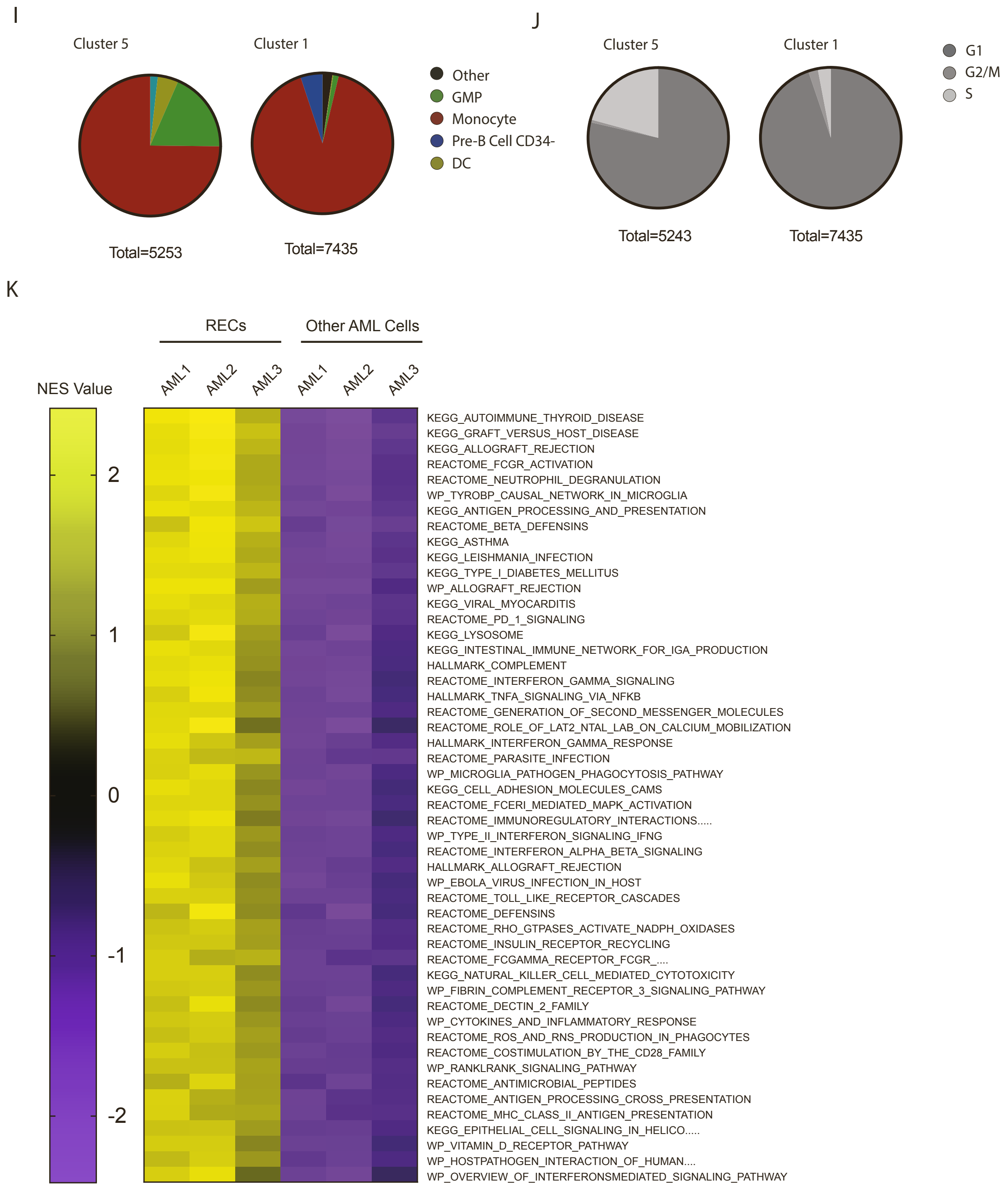

Figure S2: Functionally defined biologically relevant timepoints in AML1 and AML2 reveals transcriptionally defined cell clusters 5 and 1, respectively. Related to Figure 2. **A** Illustration of the increase in progenitor frequency (light grey line) representing leukemic regeneration and the decrease in disease burden (dark grey line) representing a state of cytoreduction. **B** Total # of AML Cells (%hCD45 * total cells harvested) and progenitor frequency (CFUs / cells seeded) from sorted CD33+ hCD45+ cells of AML1 PDX models over a AraC time course (students t test). **C** Total # of AML Cells (%hCD45 * total cells harvested) and progenitor frequency (CFUs / cells seeded) from sorted CD33+ hCD45+ cells of AML2 PDX models over a AraC time course (students t test). UMAPs of AML cells at different timepoints during leukemic regeneration in PDX models of **D** AML1 and **E** AML2. **F** Total number of cells belonging to each cluster at untreated and cytoreduced timepoints in AML1 and AML2. **G** Total number of cells belonging to each cell assignment at untreated and cytoreduced timepoints in AML1 and AML2. **H** Clusters enrichment at regeneration (Cluster % at regeneration normalized to untreated control) of AML2 biological triplicates. Composition of REC Clusters 1 and 5 by **I** cell assignment

and **J** cell cycle phase. **K** Top 50 HALLMARK KEGG REACTOME and WIKI pathways enriched (ranked by NES Values of GSEA analyses) (FDR < 0.01) in REC clusters.

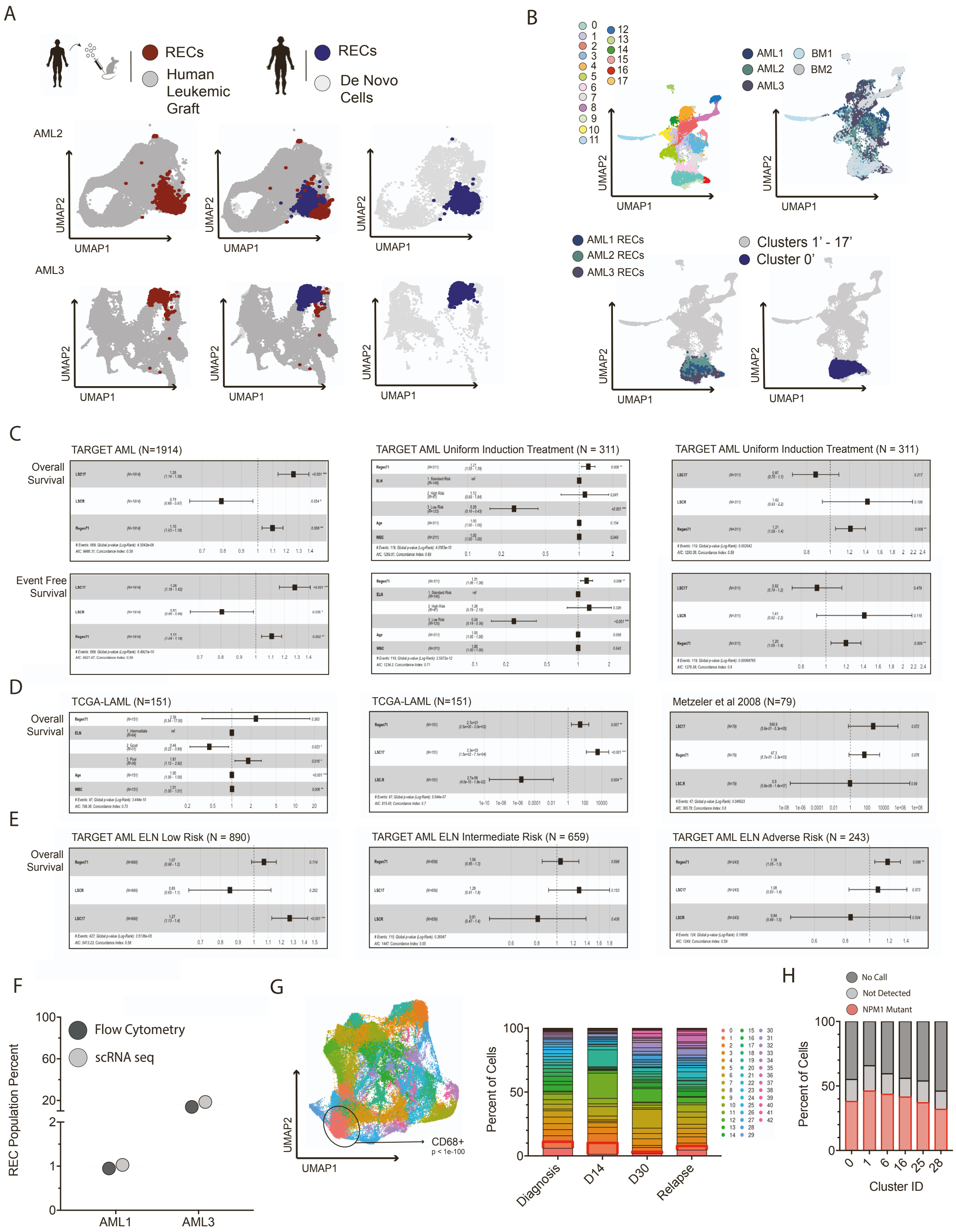

Figure S3: Related to Figure 3. RECs from AML patients demonstrate survival predictive capacity A Patient specific UMAP plots of all PDX and *de novo* cells from AML 2-3 highlighting RECs from the PDX system (dark red) and from *de novo* tissue (dark blue) **B** UMAP plots of all *de novo* samples (AML1-3, BM1-2, n = 25 996) highlighted by: Clusters 0' - 17', tissue source, AML1-3 RECs and Cluster 0' (653 DEG), respectively **C** Multivariate cox regression analyses on TARGET AML cohort (N = 1914) and a filtered version of the cohort with uniform induction treatments (N=311) assessing the association of EFS and OS with Regen71 and other molecular scores (LSC17, LSCR) and clinical covariates (WBC, ELN, age). **D** Multivariate cox regression analyses on TCGA LAML cohort (N = 151) and Metzeler 2008 (N = 79) assessing correlation to OS of Regen71 alongside other molecular scores (LSC17, LSCR) and clinical covariates (WBC, ELN, age) when available. **E** Multivariate cox regression analyses on TARGET AML cohort subdivided by ELN stratifications (N = 890, 659, and 243 for low, intermediate, and adverse risk groups, respectively) assessing correlation to EFS of the Regen71 with other molecular scores (LSC17, LSCR) **F** CD68 expression based on FC and scRNA seq on

AML specific *de novo* REC clusters in AML1 and AML3. **G** Visuals of the cell clusters from the 10 NPM1 mutated AML patients from Naldini 2023 scRNA seq dataset, both as a UMAP plot, and a bar graph of clusters over a time. CD68+ Cluster 1 highlighted in red emerged post-chemotherapy **H** Bar graph of NPM1 mutational profile of cells comprising each cluster of the Panel G scRNA data set.

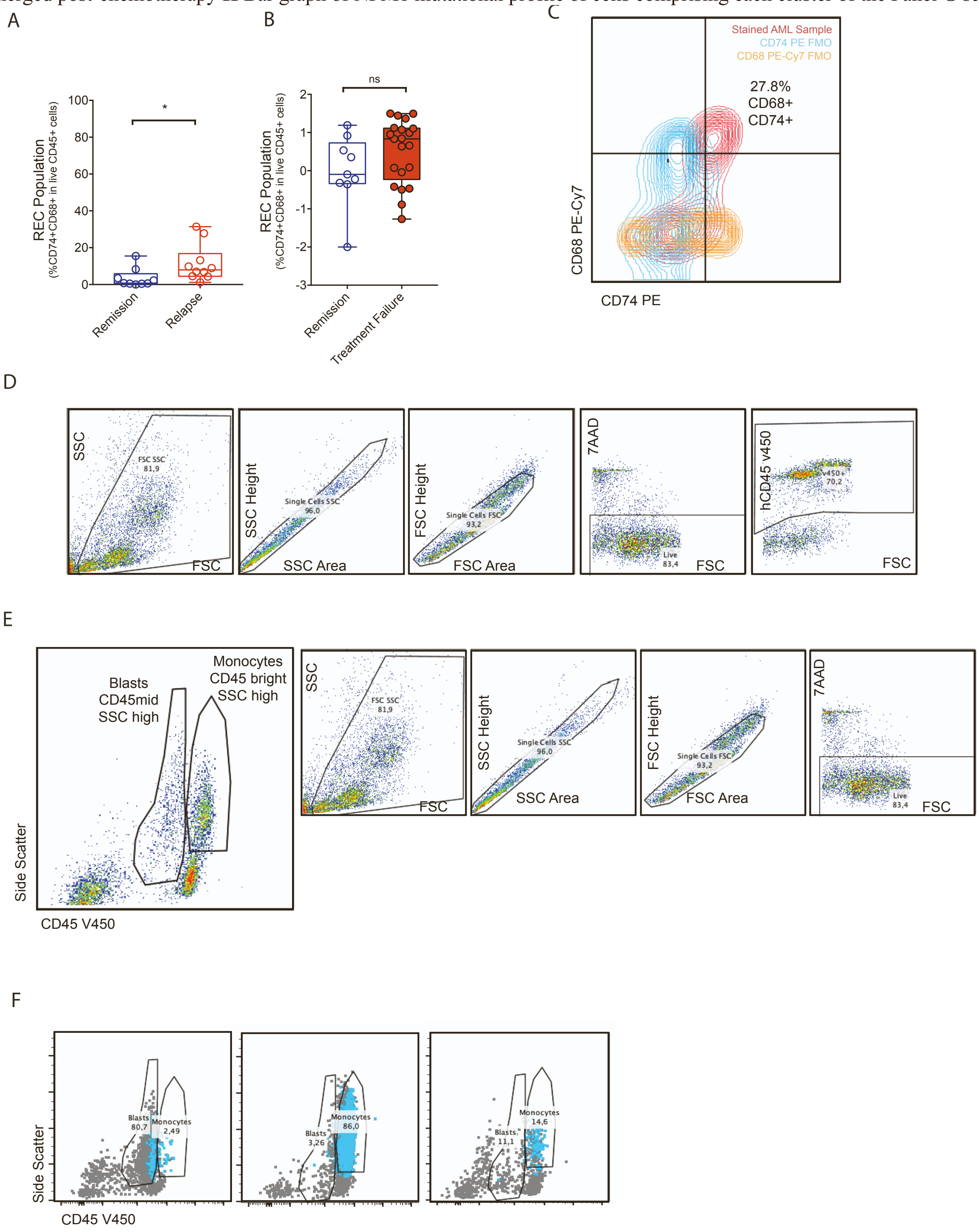

Figure S4: Flow Cytometry gating strategies of RECs, showing clinical potential. Related to Figure 4. A Bar graphs (median +/- range) of %CD74/CD68 in **A** remission (N = 9) *vs* relapse (N = 10, and **B** remission *vs.* treatment failure (N = 21) (*p < 0.05, unpaired t test) **C** Representative flow plot of CD74^{+}/CD68^{+} cells as compared to fluorescent minus one (FMO) controls **D** Representative ancestral gating strategy for CD74^{+}/CD68^{+} REC population of Panel B **E** Representative flow plot of a CD45^{bright} SSC high monocyte and blast populations of an AML sample and ancestral gating strategy **F** Three AML samples hCD45 *vs.* SSC flow

cytometry gates with $CD74^+/CD68^+$ population backgated (light blue). Location of $CD74^+/CD68^+$ cells is interpatient and interpatient heterogenous.

Table S2: ddPCR Primer Details specific to AML patient mutations. Related to Figure 5

Gene Name	AA Mutation	CDS Mutation	Mutation Type	Genomic Mutation ID	Legacy Identifier	Probe ID
IDH2	p.R172K	c.515G>A	Substitution, missense	COSV57468734	COSM33733	IDH2_515_ ANWC34M
NPM1 TCTG	p.W288Cfs*12	c.860_863dup	Insertion, frameshift	COSV51542664	COSM17559	NPM1c.860_863dupTCTG
TP53c.842A>T	p.D281V	c.842A>T	Substitution, missense	COSV52815868	COSM45729	TP53c842A-T_AN33JRA

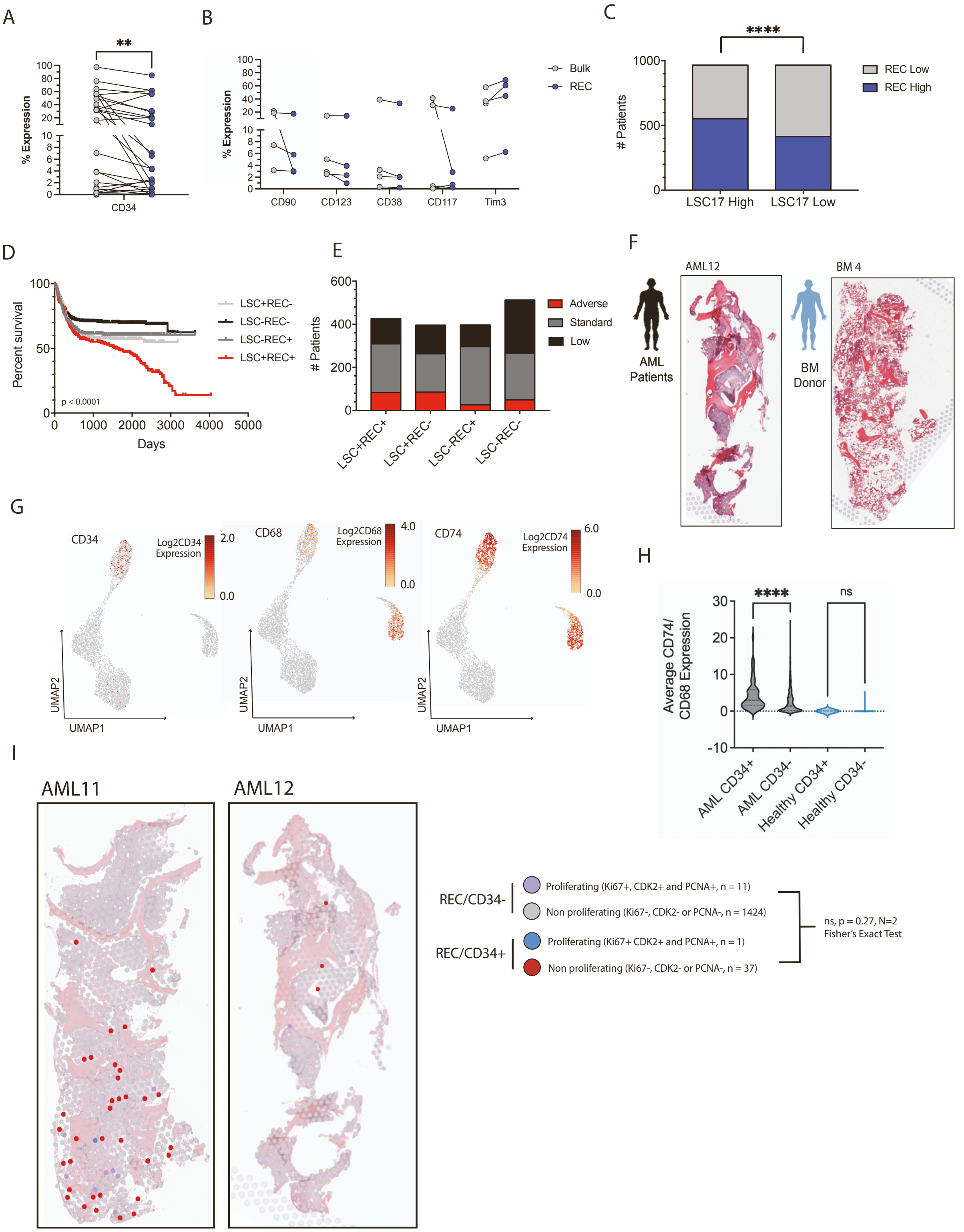

Figure S5: RECs demonstrate no stemness gene expression, contribute to OS prediction, and co-localize to CD34^{+} cells but not via proliferation, within leukemic tissue. Related to Figure 5. A Line graph of CD34^{+} expression of AML samples (N=29) compared to the REC population. **B** Line graph of Tim3, CD90, CD117 CD38, CD123 expression of AML samples (N=4, AML3,6,38,39) compared to the REC population **C** Composition of LSC17^{+} and LSC^{-} patients by REC+/- profile. LSC and REC (Regen71) high and low were decided based on the average normalized expression of the score being above or below the median of the data set. REC^{+} samples are enriched in LSC17^{+} samples (Fisher's exact test ****p < 0.0001). **D** Survival of TARGET AML cohort (N = 1914) by Kaplan-Meier curve on the four populations subdivided by REC+/- LSC+/-. REC^{+}/LSC^{-} patients had the lowest OS (p < 0.0001). **E** The change in proportion of ELN stratifications between each REC+/- LSC+/- population (p<0.0001, chi square). **F** Whole H&E stained tissue of AML10 and BM3 used for spatial **G** UMAP plots of spatial transcriptomics data highlighting areas of CD34, CD68 and CD74 expression, respectively. **H** Violin plots of average normalized CD74 and CD68 expression of healthy and AML BM sections between CD34^{+} (n=112 AML n=13 healthy) and CD34^{-} (n=1363 AML, n=2736 healthy) spots. Panel A and E: **p<0.01, ***p<0.001 by unpaired t tests).

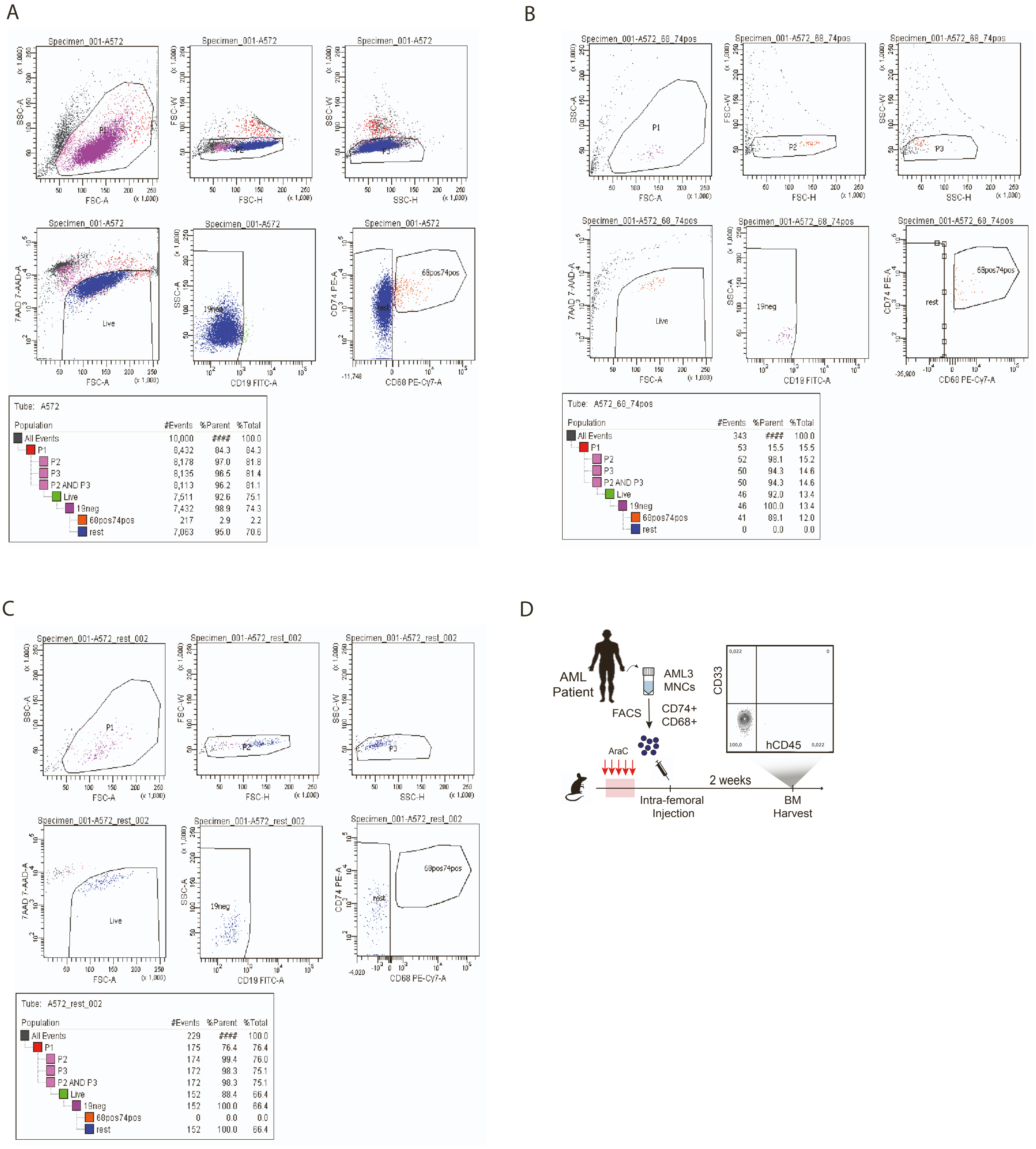

Figure S6: FACS gating strategies for REC purification which does not autonomously contribute to engraftment. Related to Figure 6. A Representative FACS gating of bulk AML cells (AML39) for the GOF and LOF experiments, where RECs (CD34^{+}/CD68^{+}) and REC depleted (either CD68^{-} or CD74^{-}) are sorted from live single cells. CD19 lymphoid cells were excluded to avoid graft vs. host disease in recipient mice. **B** Purity of sorted RECs (CD34^{+}/CD68^{+}) through the same gating system (~90%) **C** Purity of sorted REC depleted (either CD68^{-} or CD74^{-}) through the same gating system (~100%) **D** Experimental visual and representative flow plot of injected RECs into non engrafted mice to control for REC autonomous regeneration in Figure 6C. No engraftment (hCD45+/CD33+) was detected.
